# Multiomics dynamic learning enables personalized diagnosis and prognosis for pancancer and cancer subtypes

**DOI:** 10.1093/bib/bbad378

**Published:** 2023-10-26

**Authors:** Yuxing Lu, Rui Peng, Lingkai Dong, Kun Xia, Renjie Wu, Shuai Xu, Jinzhuo Wang

**Affiliations:** Department of Big Data and Biomedical AI, College of Future Technology, Peking University, Beijing, China; Department of Big Data and Biomedical AI, College of Future Technology, Peking University, Beijing, China; Department of Chemical and Biological Engineering, The Hong Kong University of Science and Technology, Hong Kong SAR, China; Department of Big Data and Biomedical AI, College of Future Technology, Peking University, Beijing, China; School of Life Sciences, Peking University, Beijing, China; Institute of Molecular Medicine, College of Future Technology, Peking University, Beijing, China; Department of Big Data and Biomedical AI, College of Future Technology, Peking University, Beijing, China

**Keywords:** multiomics Integration, dynamic learning, cancer diagnosis and prognosis, biomarker identification

## Abstract

Artificial intelligence (AI) approaches in cancer analysis typically utilize a ‘one-size-fits-all’ methodology characterizing average patient responses. This manner neglects the diverse conditions in the pancancer and cancer subtypes of individual patients, resulting in suboptimal outcomes in diagnosis and treatment. To overcome this limitation, we shift from a blanket application of statistics to a focus on the explicit recognition of patient-specific abnormalities. Our objective is to use multiomics data to empower clinicians with personalized molecular descriptions that allow for customized diagnosis and interventions. Here, we propose a highly trustworthy multiomics learning (HTML) framework that employs multiomics self-adaptive dynamic learning to process each sample with data-dependent architectures and computational flows, ensuring personalized and trustworthy patient-centering of cancer diagnosis and prognosis. Extensive testing on a 33-type pancancer dataset and 12 cancer subtype datasets underscored the superior performance of HTML compared with static-architecture-based methods. Our findings also highlighting the potential of HTML in elucidating complex biological pathogenesis and paving the way for improved patient-specific care in cancer treatment.

## INTRODUCTION

Unlocking the full potential of artificial intelligence (AI) in cancer analysis requires a departure from the conventional ‘one-size-fits-all’ approach. The concept of generalizing patient responses fails to acknowledge the complex and diverse conditions of individuals during diagnosis and treatment. This antiquated viewpoint inevitably leads to ineffective treatments, where success stories are overshadowed by disappointing outcomes. To truly revolutionize cancer analysis through AI, we must recognize and embrace the individuality of each patient.

Gone are the days when we could rely on generalized patient responses as a basis for treatment decisions. This outdated approach often leads to suboptimal outcomes, where some patients experience remarkable success while others face disappointing results. The future lies in AI-enabled personalized medicine [1], offering a solution to the uncertainties that plague cancer diagnosis and treatment planning. By shifting our focus from mere statistics and the law of averages, we can now delve into the intricate molecular-level multiomics data of each patient [2–4], pinpointing abnormalities with precision and tailoring treatment strategies accordingly [5].

This powerful tool is poised to significantly enhance the capabilities of clinicians, positioning them at the cutting edge of proactive and therapeutic interventions [6]. By harnessing the power of AI, healthcare professionals can predict the likelihood of disease onset, ensuring that intervention happens at the earliest possible stage. Beyond just detection, AI aids in tailoring treatments to individual patients, heralding the age of personalized medicine. This ensures that treatments are optimized for each patient’s unique genetic makeup and health profile, leading to more effective and fewer side effects. The integration of AI into medical practice brings with it a renewed sense of hope. As we delve deeper into the vast complexities of diseases like cancer, the promise of AI-driven solutions hints at a future where these formidable challenges can be comprehensively understood and addressed [7].

Our study proposes a highly trustworthy multiomics learning (HTML) framework (Supplementary Figure S1 available online at http://bib.oxfordjournals.org/), a multiomics dynamic learning framework that is meticulously designed to address the critical features and requirements essential for cancer analysis [8–11]. HTML offers efficiency, interpretability, and reliability in integrating multiomics data by processing each sample with data-dependent architectures and computational flows, opening new avenues for research and clinical practice. We introduced sample-adaptive feature selection and modality selection dynamic learning modules to allocate different weights to features and modalities according the input information. Inspired by biological knowledge, we devised DNA methylation-guided attention and triple contrastive learning modules within HTML, enabling us to replicate hierarchical relationships and align representations of biological components. These approaches not only enhance our understanding of various genes and data modalities but also facilitate personalized diagnosis and treatment through a sample-adaptive process. Compared with other methods, HTML stands out with its superior performance, remarkable interpretability and biologically-guided mechanisms, excelling in capturing intricate biological relationships and identifying biomarkers effectively.

To demonstrate the power of HTML, we construct a comprehensive dataset encompassing a 33-type pancancer dataset and 12 cancer subtype datasets. The results are astounding. HTML surpasses current state-of-the-art approaches, achieving a remarkable increase of 3.51% in pancancer classification accuracy and an impressive 6.8% increase in cancer subtype classification accuracy compared to previous state-of-the-art methods. Moreover, HTML proficiently classifies patients into high-risk and low-risk groups, accurately determining disease risk levels within individual cancer subtypes. Through feature dynamic weights, we identify 849 potential biomarkers across all cancer subtype datasets, many of which have already been highlighted in published articles for their significance in cancer research.

In the spirit of promoting collaboration and facilitating further scientific advancement, we have made the complete HTML package available online. This package includes tutorials, demo cases, training data, pretrained models and test results from our study, all accessible to the broader scientific community. The HTML package can be found at https://github.com/YuxingLu613/HTML.

## RESULTS

### A highly trustworthy multiomics learning model

**Figure 1:**
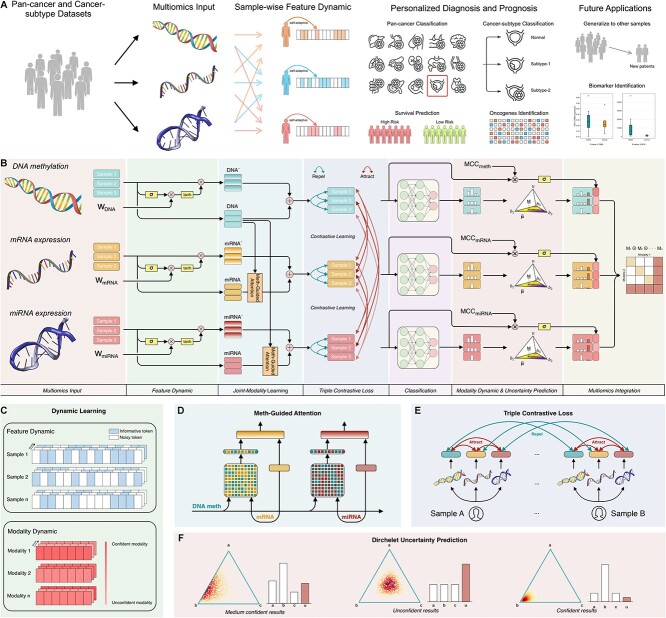
Framework of HTML. (**a**) Illustration and application of our work. (**b**) The overall pipeline of the HTML model. HTML is an end-to-end framework that integrates multiomics data to perform cancer diagnosis and prognosis. (**c**) Dynamic learning on both features and modalities. The same feature may play diverse roles across samples, and it is crucial to allocate a samplewise adaptive weight to each feature rather than relying on a fixed weight vector. Similarly, varying weights should be assigned to modalities to indicate their distinct contributions to the final results. (**d**) DNA methylation-guided attention mechanism. DNA methylation can affect the expression levels of mRNA and miRNA. This guided attention mechanism reflects the interrelationships among different modalities and outputs a coordinated representation that integrates the biological information among modalities. (**e**) Triple contrastive learning module. Multiomics learning tasks are typically conducted in an unsupervised learning setting, where the data in each modality must be subject to the overall label of the sample. In this module, modality embeddings from a given sample are considered positive pairs, while those from different samples are considered negative pairs. This approach effectively aligns the representations of different modalities, facilitating the integration of multiple data sources. (**f**) Dirichlet uncertainty prediction. If the probability distribution of a classification result is evenly distributed, it may indicate that the model has a limited capacity to differentiate between various categories. Conversely, when the probability distribution of a specific label is significantly high, it suggests that the model has a higher degree of confidence in its classification result and a more precise prediction. The contribution of each module in HTML was comprehensively addressed through the ablation study in Supplementary Table 4.

In this paper, we present HTML, a highly trustworthy multiomics learning model explicitly designed for multiomics classification tasks (as depicted in Figure 1b). The proposed framework comprises five primary modules:

- An inner-omics feature dynamic module that self-adaptively selects informative features (Figure 1c),
- An interomics DNA methylation guided attention module that appropriately integrates complementary information among diverse modalities (Figure 1d),
- A triple contrastive learning module that aligns the features of diverse modalities (Figure 1e),
- A modality dynamic module that quantifies the relative contribution of each modality toward the final classification results (Figure 1f),
- An uncertainty module for prediction and integration employs robust statistical tools (Dirichlet distribution and Dempster–Shafer theory) to provide reliable predictions and enhance the explainability of the model (Figure 1f).

When all five modules of the proposed HTML architecture are integrated with each other, the resulting framework offers both high performance and significant explainability, making it an ideal multiomics integration model for clinical applications such as cancer diagnosis and survival analysis.

HTML is an end-to-end model that can effectively improve the performance of multiomics classification tasks, enabling personalized diagnosis and treatment and facilitating the discovery of novel biomarkers. Our extensive research has led us to firmly believe that HTML constitutes the foremost approach to multiomics integration in biomedical data classification, characterized by high performance, trustworthiness and sensitivity to individual variations.

### Datasets and experimental settings

#### Dataset

We constructed a large-scale pancancer classification dataset of 33 different cancer types and 12 cancer subtype classification datasets from TCGA to showcase the effectiveness of HTML (Methods). Three types of omics data (DNA methylation (DNA meth), mRNA expression (mRNA), and micro-RNA expression (miRNA) except for the pancancer dataset) were used to enable multiomics integration and classification, leading to the elaboration of complementary information pertaining to cancers.

In our experiments, only the samples that matched omics data were included in our study (Supplementary Tables 1–3 available online at http://bib.oxfordjournals.org/). In light of the potential impact of noise and redundant features on the overall quality, we conducted preprocessing and feature preselection on each type of omics data independently. Supplementary Tables 2 and 3 (available online at http://bib.oxfordjournals.org/) display the precise feature quantity before and after preprocessing.

#### Experimental settings

We conducted a comparative analysis of the classification accuracy of HTML in relation to preexisting multiomics integration algorithms (Table 1 and Supplementary Table 4 available online at http://bib.oxfordjournals.org/) and performed a comprehensive set of ablation studies (Supplementary Table 5 available online at http://bib.oxfordjournals.org/) to demonstrate the crucial roles played by distinct components in HTML. The hyperparameter selection comparison for each dataset is shown in Supplementary Table 6 available online at http://bib.oxfordjournals.org/. To provide a more valid estimate of HTML’s performance, we executed a 5-fold cross-validation and subsequently reported the mean and standard deviation (SD) of the results on the validation fold.

**Table 1:** Performance of HTML under different cancer subtypes. To evaluate the effectiveness of HTML, we conducted experiments on 12 different datasets from TCGA and compared it with ten prevalent multiomics classification methods. Our results show that HTML outperforms all other methods in terms of accuracy, F1-macro, and F1-weighted scores on all datasets and achieves the best AUROC and AUPRC scores on most datasets. To ensure the reliability of our results, we applied 5-fold validation and calculated the mean value and standard deviation of each metric for each method.

Traditional ML methods																										
	NB					LR					SVM					RF					XGBoost					
Dataset	Accuracy	F1-macro	F1-weighted	AUC score	PRC score	Accuracy	F1-macro	F1-weighted	AUC score	PRC score	Accuracy	F1-macro	F1-weighted	AUC score	PRC score	Accuracy	F1-macro	F1-weighted	AUC score	PRC score	Accuracy	F1-macro	F1-weighted	AUC score	PRC score	
COADREAD	65.36 (3.27)	61.62 (2.96)	67.96 (2.69)	68.26 (0.86)	74.44 (0.69)	86.21 (4.40)	74.49 (6.45)	81.76 (4.79)	77.80 (7.41)	83.75 (4.98)	76.80 (3.35)	45.88 (4.06)	67.53 (5.18)	51.19 (1.65)	73.12 (5.13)	80.11 (2.98)	64.39 (5.84)	76.94 (4.06)	62.74 (4.51)	74.75 (7.19)	82.13 (4.38)	73.45 (6.19)	83.20 (5.00)	76.11 (6.21)	83.15 (4.54)	
ESCA	89.61 (5.88)	89.40 (5.78)	89.62 (5.87)	90.09 (5.56)	92.50 (3.88)	93.99 (3.54)	93.79 (3.58)	94.02 (3.54)	94.33 (3.72)	95.46 (2.64)	93.99 (3.54)	93.82 (3.55)	94.03 (3.53)	94.52 (3.52)	95.49 (2.61)	95.08 (3.01)	94.95 (2.99)	95.10 (3.00)	95.54 (2.92)	96.37 (2.11)	92.91 (5.60)	92.72 (5.57)	92.93 (5.59)	93.37 (5.41)	94.81 (3.82)	
GBMLGG	51.87 (3.80)	51.15 (2.94)	53.05 (3.42)	64.17 (2.02)	60.76 (2.00)	52.84 (7.71)	46.70 (6.50)	50.51 (8.62)	62.51 (4.50)	55.83 (5.15)	61.24 (7.14)	47.72 (5.60)	53.49 (8.76)	67.11 (3.08)	63.18 (6.87)	57.73 (5.66)	49.35 (4.43)	53.94 (6.86)	65.52 (2.82)	58.52 (2.27)	56.75 (4.52)	50.32 (3.12)	54.57 (4.68)	65.03 (2.26)	58.69 (1.87)	
SARC	64.97 (2.15)	63.76 (3.27)	65.98 (2.37)	74.29 (2.56)	71.18 (3.84)	82.90 (2.75)	81.43 (2.36)	82.62 (2.85)	85.83 (2.24)	85.46 (1.57)	77.04 (3.53)	73.56 (5.01)	76.22 (4.26)	80.59 (3.88)	80.73 (3.59)	78.21 (2.18)	75.46 (2.94)	77.87 (2.18)	81.71 (1.64)	81.20 (2.85)	75.85 (5.20)	72.85 (5.87)	75.45 (5.29)	79.71 (3.79)	77.93 (5.47)	
STAD	53.37 (0.68)	53.02 (0.50)	53.14 (0.89)	54.33 (1.61)	66.06 (1.80)	62.53 (4.66)	61.78 (4.49)	62.47 (4.71)	62.07 (4.48)	71.47 (3.27)	56.86 (5.78)	54.90 (6.26)	55.67 (6.69)	56.30 (5.46)	67.48 (4.35)	60.11 (3.88)	59.47 (4.04)	59.90 (4.52)	60.33 (3.70)	70.32 (2.57)	63.60 (7.15)	62.91 (7.69)	63.41 (7.51)	63.66 (7.53)	72.78 (5.61)	
STES	90.17 (3.15)	90.00 (3.17)	90.14 (3.15)	89.88 (3.16)	92.54 (2.36)	94.55 (3.30)	94.49 (3.32)	94.55 (3.30)	94.56 (3.40)	95.88 (2.53)	95.11 (3.53)	95.04 (3.60)	95.11 (3.53)	95.16 (3.60)	96.30 (2.69)	93.45 (1.43)	93.38 (1.51)	93.46 (1.43)	93.57 (1.60)	95.10 (1.17)	91.83 (4.64)	91.74 (4.67)	91.84 (4.64)	91.84 (4.67)	93.86 (3.55)	
THCA	77.20 (6.76)	70.16 (5.32)	78.62 (6.01)	74.39 (6.25)	77.05 (4.16)	81.40 (5.13)	67.55 (7.10)	80.06 (5.55)	66.00 (6.69)	73.37 (5.88)	81.60 (4.51)	64.69 (6.86)	79.17 (4.86)	62.70 (5.68)	72.83 (7.41)	83.80 (2.59)	71.02 (7.17)	82.58 (2.82)	69.46 (7.34)	76.46 (4.95)	82.20 (4.76)	69.40 (6.75)	81.20 (4.54)	67.93 (6.78)	74.75 (5.35)	
UCEC	71.40 (4.24)	47.62 (6.00)	72.50 (4.04)	67.59 (4.20)	54.44 (5.35)	83.49 (5.03)	53.40 (4.61)	82.61 (5.57)	71.66 (4.56)	59.61 (9.93)	85.12 (4.30)	54.39 (4.13)	82.86 (5.22)	71.41 (4.61)	73.75 (3.45)	83.95 (4.53)	52.94 (4.10)	81.15 (5.35)	68.69 (4.51)	72.77 (3.09)	84.65 (3.80)	54.50 (2.79)	82.63 (4.92)	71.47 (4.12)	67.42 (8.91)	
BRCA	74.97 (3.44)	74.16 (4.71)	76.42 (2.89)	87.33 (3.09)	79.09 (3.93)	78.63 (2.87)	74.72 (4.96)	78.86 (3.26)	83.57 (2.87)	77.66 (4.44)	82.06 (3.80)	78.77 (5.77)	82.32 (3.75)	86.57 (3.40)	82.20 (5.08)	74.06 (3.22)	66.75 (3.87)	73.43 (3.37)	78.78 (3.25)	70.24 (3.35)	74.97 (3.06)	68.23 (2.49)	74.67 (3.19)	80.37 (1.93)	71.40 (2.35)	
LGG	63.35 (3.54)	61.24 (4.32)	61.20 (4.01)	63.89 (4.59)	74.93 (4.01)	66.79 (4.47)	66.61 (4.45)	66.76 (4.46)	66.71 (4.59)	75.04 (3.50)	68.50 (5.27)	68.00 (5.37)	68.07 (5.24)	68.74 (5.90)	77.24 (5.04)	66.59 (4.39)	66.32 (4.39)	66.45 (4.38)	66.58 (4.54)	75.09 (3.51)	65.83 (8.01)	65.63 (8.01)	65.78 (7.99)	65.77 (8.18)	74.38 (6.25)	
KIPAN	85.15 (2.10)	82.90 (3.15)	85.35 (1.94)	89.39 (2.20)	86.41 (2.60)	95.19 (1.37)	94.02 (1.45)	95.21 (1.37)	96.26 (1.32)	94.95 (1.31)	93.78 (2.14)	92.24 (2.46)	93.80 (2.09)	94.66 (1.44)	93.34 (2.05)	91.66 (2.55)	90.46 (2.88)	91.67 (2.53)	93.18 (1.79)	91.98 (2.49)	93.78 (1.95)	92.88 (2.17)	93.80 (1.92)	95.29 (1.61)	94.09 (1.88)	
ROSMAP	66.37 (5.48)	66.10 (5.62)	66.12 (5.61)	66.92 (5.80)	75.56 (4.45)	67.80 (4.26)	67.47 (4.66)	67.55 (4.57)	68.19 (4.60)	76.44 (3.33)	67.52 (4.00)	67.22 (4.22)	67.28 (4.26)	67.94 (4.17)	76.27 (3.00)	66.94 (5.45)	66.53 (6.03)	66.58 (5.85)	67.49 (6.44)	76.02 (4.98)	68.68 (5.01)	68.46 (5.02)	68.62 (5.14)	68.72 (5.21)	76.56 (3.80)	
**Deep Neural Network**																										
	**MOGONET (2021)**					**MMDynamic (2022)**					**EMOGI (2021)**					**Subtype-DCC (2023)**					**TMC (2022)**					
Dataset	Accuracy	F1-macro	F1-weighted	AUC score	PRC score	Accuracy	F1-macro	F1-weighted	AUC score	PRC score	Accuracy	F1-macro	F1-weighted	AUC score	PRC score	Accuracy	F1-macro	F1-weighted	AUC score	PRC score	Accuracy	F1-macro	F1-weighted	AUC score	PRC score	Uncertainty
COADREAD	79.79 (6.39)	60.53 (9.36)	74.61 (9.26)	73.21 (11.09)	70.77 (7.36)	76.48 (6.03)	43.27 (1.96)	66.42 (8.14)	73.94 (5.62)	69.74 (3.87)	79.20 (6.83)	58.73 (14.22)	73.94 (10.58)	73.24 (6.19)	68.89 (4.78)	82.21 (4.28)	64.18 (14.17)	78.05 (7.69)	77.73 (7.63)	74.51 (7.14)	77.38 (6.49)	49.90 (7.16)	69.48 (9.23)	77.80 (2.71)	73.11 (2.43)	65.08 (1.92)
ESCA	94.35 (1.35)	94.33 (1.36)	94.35 (1.35)	94.74 (1.68)	94.46 (2.27)	96.68 (4.08)	96.67 (4.10)	96.69 (4.06)	98.75 (1.83)	96.27 (2.01)	96.70 (2.70)	96.67 (2.72)	96.70 (2.69)	98.51 (2.08)	98.10 (2.91)	96.17 (2.77)	96.11 (2.80)	96.15 (2.78)	98.73 (1.10)	98.77 (1.10)	97.36 (2.17)	97.35 (2.18)	97.36 (2.17)	97.87 (1.61)	96.68 (2.01)	65.04 (3.95)
GBMLGG	54.60 (5.76)	42.35 (5.59)	47.41 (7.42)	72.51 (2.07)	58.09 (2.51)	57.14 (4.05)	46.19 (5.85)	50.95 (5.20)	70.69 (5.22)	54.50 (5.12)	60.26 (4.84)	45.81 (2.71)	51.84 (5.67)	71.80 (2.86)	57.26 (4.41)	58.11 (7.44)	44.80 (4.64)	50.47 (7.63)	69.48 (4.78)	52.90 (5.24)	54.76 (9.92)	39.89 (9.30)	45.20 (11.01)	69.05 (9.86)	53.46 (9.97)	75.53 (4.18)
SARC	83.70 (6.50)	82.70 (5.81)	83.61 (6.62)	91.85 (2.92)	85.83 (2.33)	78.27 (8.19)	74.93 (13.74)	75.70 (12.01)	91.59 (3.59)	86.34 (6.41)	78.21 (7.73)	70.24 (11.74)	75.39 (9.32)	91.90 (4.12)	84.19 (8.14)	79.00 (7.67)	75.14 (10.48)	76.74 (9.27)	91.66 (3.60)	86.26 (5.59)	81.31 (2.72)	80.66 (3.26)	80.67 (3.46)	92.72 (1.36)	88.42 (2.62)	64.24 (0.81)
STAD	63.08 (2.84)	56.51 (7.82)	58.19 (5.12)	63.71 (6.17)	63.68 (5.52)	64.42 (3.17)	59.81 (9.49)	61.47 (6.80)	64.26 (3.26)	63.24 (3.46)	62.28 (4.67)	55.84 (8.08)	57.75 (6.77)	64.09 (4.16)	63.25 (3.15)	64.70 (5.50)	60.93 (7.06)	61.70 (7.28)	64.12 (5.70)	64.59 (5.41)	60.38 (2.42)	49.03 (7.92)	51.84 (6.51)	61.73 (4.03)	61.38 (3.42)	52.77 (5.39)
STES	89.10 (14.08)	84.21 (23.33)	86.18 (19.70)	82.42 (29.99)	84.65 (23.82)	94.02 (4.32)	93.78 (4.51)	94.03 (4.32)	97.63 (2.27)	97.48 (2.40)	89.11 (9.49)	88.72 (9.69)	88.79 (10.06)	98.28 (2.27)	97.73 (3.39)	98.33 (2.22)	98.28 (2.29)	98.33 (2.24)	99.55 (0.63)	99.52 (0.70)	88.60 (6.66)	88.21 (6.73)	88.51 (6.66)	97.34 (2.22)	96.98 (2.64)	46.96 (2.55)
THCA	83.20 (6.05)	62.09 (13.36)	78.30 (9.02)	84.53 (4.11)	77.05 (3.95)	80.40 (4.22)	50.28 (7.29)	72.92 (6.70)	83.50 (3.43)	75.94 (2.14)	86.00 (2.28)	75.03 (6.10)	85.07 (2.57)	85.73 (2.78)	78.88 (4.68)	86.20 (1.17)	75.38 (3.97)	85.23 (1.70)	85.33 (2.47)	79.58 (2.11)	83.20 (3.43)	62.21 (7.06)	78.97 (5.01)	84.51 (4.73)	78.22 (5.15)	36.05 (1.63)
	**MOGONET (2021)**					**MMDynamic (2022)**					**EMOGI (2021)**					**Subtype-DCC (2023)**					**TMC (2022)**					
UCEC	86.51 (5.38)	54.82 (7.78)	83.21 (7.06)	83.80 (5.08)	65.53 (1.79)	81.16 (4.44)	47.25 (7.34)	76.51 (6.59)	84.79 (3.30)	63.43 (3.54)	84.88 (4.65)	52.45 (8.22)	81.40 (5.86)	85.47 (4.69)	67.20 (5.71)	84.19 (5.48)	55.18 (6.29)	81.44 (6.90)	86.91 (0.95)	64.44 (1.92)	79.53 (6.18)	45.70 (9.72)	74.40 (10.25)	85.78 (3.13)	62.97 (3.34)	43.71 (1.07)
BRCA	64.69 (7.26)	38.57 (11.43)	55.57 (9.94)	89.37 (2.16)	71.48 (6.75)	57.60 (6.77)	26.82 (4.07)	44.82 (7.05)	85.56 (3.43)	61.41 (3.89)	63.54 (2.81)	34.24 (5.35)	51.86 (5.09)	88.59 (1.51)	68.41 (4.92)	67.43 (4.47)	42.52 (6.32)	59.44 (7.70)	90.80 (1.63)	76.15 (4.14)	57.94 (6.45)	25.01 (8.41)	43.65 (7.56)	88.59 (2.68)	67.22 (7.93)	48.18 (0.67)
LGG	75.38 (2.00)	74.56 (2.27)	74.98 (2.06)	76.19 (3.13)	76.23 (2.28)	85.87 (4.30)	74.67 (14.65)	84.29 (5.73)	95.33 (3.86)	87.08 (11.03)	69.84 (3.38)	67.42 (3.28)	67.89 (3.87)	75.90 (3.61)	76.78 (3.76)	71.57 (5.53)	69.51 (8.57)	69.94 (8.29)	78.36 (2.26)	78.07 (1.43)	66.05 (5.42)	65.02 (5.64)	65.14 (5.68)	70.44 (5.42)	70.64 (5.54)	48.38 (6.84)
KIPAN	94.20 (2.63)	87.00 (12.61)	93.56 (3.71)	98.92 (0.43)	97.01 (1.84)	67.37 (5.29)	63.96 (8.37)	64.88 (7.72)	75.24 (3.56)	74.89 (3.64)	93.06 (3.23)	90.92 (3.78)	93.01 (3.32)	98.75 (0.51)	95.86 (2.71)	94.48 (3.31)	92.91 (3.58)	94.50 (3.28)	98.97 (0.31)	95.69 (3.39)	89.80 (5.52)	82.96 (12.35)	88.79 (6.52)	98.38 (0.90)	94.50 (3.38)	43.62 (0.68)
ROSMAP	71.52 (2.29)	70.29 (2.08)	70.69 (2.15)	75.36 (3.09)	76.88 (2.83)	56.99 (5.32)	46.52 (11.58)	47.82 (10.57)	66.69 (6.48)	67.15 (6.12)	66.99 (7.05)	66.13 (7.08)	66.20 (7.23)	73.27 (6.40)	73.91 (5.91)	62.43 (9.86)	55.71 (17.19)	55.63 (17.13)	76.63 (3.24)	77.69 (2.94)	58.11 (3.16)	49.27 (7.13)	50.74 (6.33)	65.17 (12.62)	67.40 (11.03)	48.85 (4.84)
																										
HTML (Ours)																										
Dataset		Accuracy		F1-macro		F1-weighted		AUC score		PRC score		Uncertainty														
COADREAD		86.43 (6.30)		76.87 (10.33)		84.79 (7.97)		89.20 (5.19)		86.00 (9.92)		58.76 (1.13)														
ESCA		98.81 (2.07)		98.79 (2.10)		98.80 (2.08)		98.93 (1.69)		97.27 (2.70)		53.88 (5.06)														
GBMLGG		65.16 (3.84)		55.93 (4.83)		59.91 (6.26)		70.95 (3.69)		69.34 (2.09)		71.69 (0.40)														
SARC		88.35 (2.65)		87.95 (1.77)		88.34 (2.70)		94.76 (1.72)		88.70 (3.77)		48.49 (1.33)														
STAD		68.48 (4.13)		67.18 (4.73)		67.84 (4.64)		68.73 (3.04)		64.00 (2.02)		47.93 (1.14)														
STES		98.90 (1.34)		98.86 (1.40)		98.91 (1.33)		99.55 (0.76)		97.85 (1.75)		53.75 (13.66)														
THCA		85.40 (1.85)		72.98 (2.89)		84.00 (1.66)		85.12 (3.27)		77.49 (3.12)		34.60 (0.52)														
UCEC		87.91 (3.57)		57.77 (3.18)		85.61 (3.81)		82.88 (2.50)		65.32 (6.13)		20.62 (0.44)														
BRCA		88.21 (3.57)		88.73 (3.16)		85.86 (4.27)		92.26 (1.47)		86.33 (3.55)		44.34 (0.33)														
LGG		77.10 (2.14)		76.94 (2.26)		77.10 (2.07)		79.92 (1.48)		79.50 (1.65)		36.90 (0.93)														
KIPAN		96.71 (1.46)		95.76 (1.99)		96.22 (1.44)		99.16 (0.43)		96.84 (3.02)		37.86 (0.65)														
ROSMAP		84.91 (2.99)		84.97 (2.97)		89.88 (3.00)		87.48 (3.22)		88.59 (2.96)		61.78 (1.84)														

We employed a battery of metrics for the evaluation of the selected methods: accuracy, average F1 score weighted by support (F1-weighted), macroaveraged F1 score (F1-macro), average area under the receiver operating characteristic curve (AUROC) and average area under the precision-recall curve (AUPRC) for each classification task. It is worth mentioning that we have incorporated an uncertainty metric in HTML to convey the model’s level of confidence in the classification results. In all of our experiments, we conducted the research on a single Tesla V100 GPU.

### HTML outperforms existing methods in pancancer and cancer subtype diagnosis

To evaluate HTML’s performance in cancer-subtype classification, we compared the classification performance of HTML with the following ten existing classification algorithms (five are traditional machine learning models and five are deep neural networks):

- Naive Bayes (NB) [12].
- Linear regression (LR) [13].
- Support vector machine classifier (SVM) [14].
- Random forest classifier (RF) [15].
- Gradient tree boosting-based classifier (XGBoost) [16].
- Multi-Omics Graph cOnvolutional NETworks (MOGONET) [8].
- Multimodal Dynamics (MMDynamics) [17].
- Explainable Multiomics Graph Integration (EMOGI) [9].
- Subtype-DCC [18].
- Trusted Multi-View Classification (TMC) [19].

All the above methods were trained with the same preprocessed data. All the evaluation metrics of the cancer subtype classification tasks are shown in Table 1, and all the evaluation metrics of the pancancer classification tasks are shown in Supplementary Table 4 available online at http://bib.oxfordjournals.org/.

As shown in Figure 1a and Supplementary Figure 2 available online at http://bib.oxfordjournals.org/, HTML exhibits outstanding performance in the 33-class pancancer classification task, achieving an overall accuracy of 93.34 and an F1-weighted score of 92.22. Notably, we observed that the majority of misclassifications occurred within the same cancer meta-type, such as colon adenocarcinoma (COAD) and rectal adenocarcinoma (READ), both of which are malignancies that manifest in the large intestine. Furthermore, a subset of ESCA samples is erroneously classified as HNSC, implying a potential correlation between these cancer subtypes.

With regard to cancer subtype classification, it is evident that HTML outperformed other methods for most cancer subtype classification datasets. However, some exceptional cases emerged. For instance, the AUROC of MOGONET exceeded that of HTML on the GBMLGG dataset by 1.56%, but HTML still performed better than MOGONET regarding all other metrics. Moreover, although HTML exhibited incompetence with SVM and MOGONET in the STAD and KIPAN datasets, it surpassed all other models across the remaining metrics. Notably, the data imbalance in the UECE dataset, where the largest subtype contains ~15 times more samples than the smallest subtype, may inherently introduce instability into the AUROC and AUPRC results, which may explain the relatively weak results of HTML in the UECE dataset. Overall, HTML demonstrated a strong classification performance across the 12 cancer-subtype classification tasks while surpassing other state-of-the-art methods in all accuracy, F1-macro, and F1-weighted metrics.

Regarding individual datasets, our HTML model achieved high cancer subtype classification results in some relatively straightforward binary classification tasks, such as ESCA, STES and KIPAN. However, the model faced challenges in tougher classification tasks such as GBMLGG and STAD. In the GBMLGG dataset, the model may have experienced difficulty distinguishing and classifying the three subtypes of astrocytoma (ASTRO), oligodendroglioma (ODG) and oligoastrocytoma (OA) due to their common origin from glial cells of the brain and spinal cord. Similarly, in the STAD dataset, there existed ambiguous relationships between the labels adenocarcinoma (ADC) and intestinal adenocarcinoma (IAC), wherein IAC identifies a distinct ADC subtype found in the mucous membrane of the intestines forming a subset of ADCs. This complex label relationship may have contributed to the model’s difficulties in accurately classifying these subtypes.

### HTML predicts accurate cancer subtype prognosis

**Figure 2:**
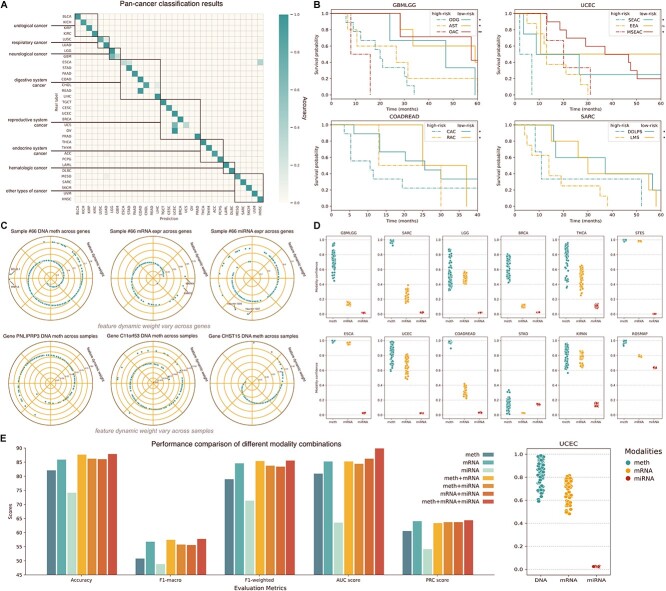
HTML is a prominent model in multiomics learning. **(a**) We visualized the classification confusion matrix of HTML on the pancancer dataset, and the results demonstrate that HTML exhibits exceptional performance in pancancer classification. (**b**) We performed survival prediction tasks on the GBMLGG, UCEC, COADREAD and SARC datasets to identify the high-risk and low-risk groups. The predicted risk groups are divided by the cancer subtypes and visualized by Kaplan–Meier curves. The dashed line represents the high-risk group, and the solid line represents the low-risk group. The *P*-value is annotated in the graph, where ^*^ means *P*-value $\le 0.05$, ^*^^*^ means *P*-value $\le 0.01$, and ‘ns’ indicates not significant. (**c**) We illustrate the distribution of feature dynamic weights under different conditions. We randomly selected the 66th sample’s feature dynamic weight in different modalities from the ESCA dataset (upper 3 charts) and found that their distribution was significantly different. The same phenomena were also found in the same feature dynamic weights across samples (lower 3 charts). (**d**) We extracted the modality dynamic weights’ distribution in all datasets and determined that the modality dynamic weights are all related to the data distribution in the specific sample. (**e**) We tested all possible multiomics input combinations and examined their corresponding classification performance.

HTML is an adaptable framework that can be proficiently utilized for cancer prognosis tasks. To elucidate this point, we chose four datasets, namely, GBMLGG, UCEC, COADREAD and SARC, to predict patient cancer development across diverse cancer subtypes. The Kaplan–Meier curves for risk prediction in these patients are presented in Figure 2b.

Our analysis enabled us to effectively differentiate between high-risk and low-risk cohorts for each cancer subtype across all datasets. The low-risk group is represented by a solid line, while the high-risk group is denoted by a dotted line. For example, in the GBMLGG dataset, patients categorized as low-risk for oligodendroglioma (ODG), astrocytoma (AST) and oligoastrocytoma (OAC) demonstrated significantly superior cancer progression outcomes relative to their high-risk counterparts, and this difference was most pronounced in OAC subtypes, where the *P* value between low-risk and high-risk groups was <0.01. Similarly, in the UCEC dataset, patients with serous endometrial adenocarcinoma (SEAC) and mixed serous and endometrioid (MSEAC) were effectively stratified based on cancer progression risk. However, the prognostic performance for endometrioid endometrial adenocarcinoma (EEA) patients is not statistically significant, which is possibly due to a dearth of available samples. Moreover, the results from the COADREAD and SARC datasets further reinforce HTML’s prognostic capabilities. Taken together, these findings provide compelling evidence of HTML’s ability to accurately predict cancer progression in patients.

### HTML enables personalized diagnosis and treatment

Intuitively, (i) the variation of impact for different features within a given sample on the model’s results should be distinct. (ii) The variation in attention given to a particular feature across different samples should be distinct. (iii) The variation in attention given to each feature during different training stages for a given sample should be distinct. Most existing encoder models learn a single set of weights for all inputs, which constrains their ability to fully explore the inherent characteristics of the features and provides understandable interpretations of the original data. To overcome this limitation, we introduced the feature dynamic concept, which adaptively learns feature dynamic weights based on the feature’s characteristics and distribution. In contrast to data preprocessing, which aims to convert raw data into an analysis-ready format by eliminating any anomalies or inconsistencies present in the dataset, feature engineering is devised to allot varying weights to different features across individual samples, enabling the model to focus intently on the most informative features of the dataset.

Based on the analysis of the multiomics data, HTML can be used not only for the diagnostic and prognostic prediction of pancancer or cancer subtypes but also for self-adaptive assignment of cancer-related genes in specific samples through dynamic learning, thereby achieving dynamic monitoring of cancer progression and providing real-time guidance for individual precision treatment. In the randomly selected 66th sample (Figure 2c), HTML assigned different dynamic weights to the genes associated with cancer subtypes. Among the genes in this sample, DNA methylation of B-cell lymphoma-2-like protein 1 (BCL2L1) and hepatocyte nuclear factor 1 homeobox A (HNF1A) were given the highest feature dynamic weights. As a member of the antiapoptotic Bcl-2 family, BCL2L1 is hypomethylated and highly expressed in chemotherapy drug (e.g. cisplatin and paclitaxel) -resistant tumor cells in vitro and has been proven to be associated with drug resistance and recurrence of solid tumor cancer [20–23]. In contrast, aberrant hypermethylation of HNF1A downregulates the expression of UDP-glucuronosyltransferase 1A1 (UGT1A1), which remodels drug metabolism and transport pathways, locally inactivating anticancer drugs by glucuronidation in colon cancer cells [24]. In terms of mRNA expression, microtubule affinity regulating kinase 4 (MARK4) and Laminin Subunit Gamma 2 (LAMC2), with the highest dynamic weights in sample 66, may become potential high-efficiency targets for cancer treatment. MARK4 can promote the proliferation and migration of cancer cells by inhibiting Hippo signaling, the targeted inhibition of which is a strategy to treat cancers [25]. However, its absence restricts the tumorigenicity of cancer cells [26]. Upregulated expression of LAMC2, an indicator of progression in esophageal squamous cell carcinoma (ESCC), is caused by lncRNA Cancer Susceptibility 9 (CASC9) interacting with CREb-binding proteins and is common in patients [27]. LAMC2 and CASC9 have been shown to be important biomarkers for metastasis therapy and the prognosis of ESCC, confirming the potential for HTML to play a role in cancer diagnosis and treatment [28]. HTML can also dynamically evaluate miRNA expression based on multiomics data analysis. The sponging of miR-1304 has been studied in many types of cancer, and this phenomenon that is regulated by multiple circRNAs leads to cancer proliferation and invasion [29–31]. However, miR-1307 is involved in tumor development by influencing target genes, such as Forkhead box O3a (FOXO3A), SET And MYND Domain Containing 4 (SMYD4) and Disabled homolog 2-interacting protein (DAB2IP), to further promote cancer invasion [32–34]. These genes, which were given the highest dynamic weight, may be potential targets for precision cancer therapy. We also extracted feature dynamic weights from three different genes in each sample shown in Figure 2c, with variations being exhibited across different samples. Depending on the sensitivity of HTML to individual differences, cancer diagnosis and treatment will likely be more personalized and effective in the future.

Similar to feature dynamic learning, the quality and noise level of data across modalities and samples vary. Therefore, there is a need for modulated prerequisites in the contribution of each modality toward the ultimate outcome along with the model’s confidence toward each modality that aligns with data quality. As a solution, we devised a modality dynamic learning module that imparts a confidence rating for each modality. As illustrated in Figure 2d, the modality dynamic weights for different modalities across various datasets are visualized with individual data points representing each sample. Our model reveals that miRNA plays a primarily subordinate role in the classification of most datasets, with the exception of ROSMAP, which aligns with the well-established association between various miRNAs and Alzheimer’s disease. For example, Cogswell *et al.* [35] reported that the expression level of hsa-miR-423 underwent significant changes in the hippocampus and medial frontal gyrus of early- and late-stage AD patients compared with control samples. Some studies also identified an overlap in the expression patterns of specific miRNAs (hsa-miR-155, hsa-miR-126a, hsa-miR-23a, hsa-miR-34a, hsa-miR-9, hsa-miR-27a and hsa-miR-146a) in the retina of a rat model of age-related macular degeneration (AMD) (αβ intravitreal injection) and in the serum of AMD patients. These miRNAs are also recognized as potentially useful biomarkers of AD pathology [36–38].

It is worth noting that our analysis of unimodality data classification (Supplementary Table 7 available online at http://bib.oxfordjournals.org/ and Figure 2e) revealed that higher classification accuracy was achieved for mRNA expression than for DNA methylation and miRNA. This could be attributed to the fact that among these three modalities, mRNA expression is most relevant to the phenotype of the corresponding cancer. However, in our analysis of modality dynamics, we observed that weights for DNA methylation were comparatively higher than those for mRNA expression in the SARC, COADREAD and BRCA datasets and took the leading positions in all other datasets. This finding provides valuable insight into the critical upstream role that DNA methylation may play in the biological processes and pathogenesis of these cancers. It is important to note that this could also be attributed to HTML’s alignment process in the methylation-guided attention mechanism.

### HTML provides aligned representations and interpretable predictions

The various omics data, analogs to different biological processes, should adhere to the central dogma of molecular biology, whereby mRNA and miRNA expression is regulated by the DNA methylation process. Consequently, it is crucial to harmonize the representation of mRNA and miRNA expression with that of DNA methylation. In accordance with this tenet, we developed a guided attention module (Figure 1d). To exemplify the interconnectivity among genes, we randomly selected an interomics attention map (first 20 genes in each modality) from the SARC dataset of a patient afflicted with leiomyosarcoma cancer (LMS). As illustrated in Figure 1a, diverse DNA methylation spots may exhibit varying attention weights toward mRNAs and miRNAs, thus mirroring the intricate interrelationships among these genes. Moreover, we employed the concept of skip connections [39] to facilitate enhanced generalization capacity and superior performance after the guided attention module (Figure 1b).

**Figure 3:**
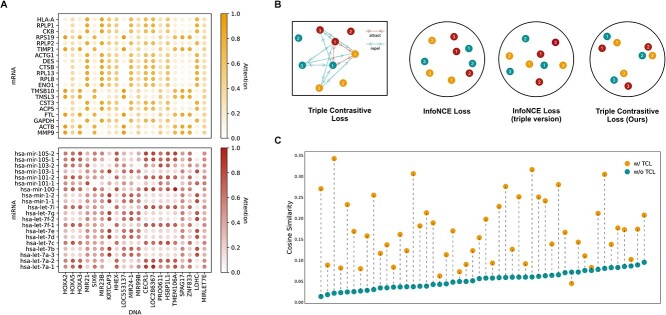
Representation alignment across different modalities. (**a**) We extracted the attention weight matrices (among the first 20 genes) from the methylation-guided attention module in the SARC dataset, which provides insights into the relationships and interactions between the genes. The upper matrix represents attention between DNA methylation and mRNA, and the lower matrix represents attention between DNA methylation and miRNA. The depth of colour in the matrix indicates the strength of these relationships. These insights can be useful in understanding the mechanisms behind gene regulation and identifying potential targets for therapeutic interventions. (**b**) We devised the triple contrastive loss (TCL) function to enhance the alignment of vector representations among different modalities within the same sample. In comparison to other contrastive learning loss functions, such as InfoNCE loss and the InfoNCE loss between triples, our TCL approach is more effective in aligning the representation of a given sample and increasing the distance between different samples. (**c**) We visualized the average cosine similarity among different modalities’ representations, and we discovered that our TCL method leads to a more aligned representation of different modalities within the same sample.

Multiomics learning incorporates a weakly supervised learning assumption, where each modality of multiomics data pertaining to a particular sample, such as DNA methylation, mRNA expression, and miRNA expression, represents a distinct biological perspective. An inherent correlation exists among the different modalities, and they can be mapped to an overall label separately. To increase the accuracy of the overall task, representation vectors for each modality of a given sample should be as close as possible. However, current contrastive learning methods [40, 41] (Figure 3c) consider little about different elements belonging to the same cluster. They tend to choose negative samples randomly from the entire dataset, which can drive away the representation of different elements of the same cluster. To address this challenge, we proposed a triple contrastive learning (TCL) method 1e. We further compared the cosine similarities among representations of different modalities with and without contrastive learning in Figure 3d. We selected the first 50 samples from the STES dataset for display. The value of each point in Figure 3d is the result of the average cosine similarity among the three modalities’ representation vectors in the sample. It can be seen that in most cases (except for one sample), the vector representations of the three modalities became more concentrated and their similarity to each other was greatly increased after being trained with TCL, where the most evident one changed from the similarity of 0.0216 to 0.3424.

The Softmax operator is widely employed in determining the classification evidential probability [42]. This involves normalizing the neural network output using $\sigma \left({z}_i\right)=\frac{e_i^z}{\sum_{k=1}^K{e}^k}$ and subsequently selecting the class with the maximum confidence probability. Nonetheless, the conventional approach to Softmax operation places inadequate focus on the overall probability distribution of classification results, with emphasis solely on the class with the highest probability [43]. Notably, the class probabilities other than the highest one can actually be informative with respect to classification outcome. Thus, considering the entire probability distribution of classification results is crucial for gaining comprehensive insight into the problem under investigation, we introduced the reliability of the classification results.

Additionally, other late fusion strategies simply concatenate the classification results for each modality or apply average pooling. HTML applies the Dempster–Shafer Theory of Evidence, which follows a trustworthy multimodal integration (TMI) rule for multimodal fusion and uncertainty calculation. The goal of TMI can be summarized in three points: (i) A modality’s classification result should be referenced to its confidence score; (ii) When the uncertainty of all modalities is high, the final prediction must be of low certainty, and vice versa; (iii) The final prediction and uncertainty score should be considered together and dynamically change according to each modality’s predicted outcome.

**Figure 4:**
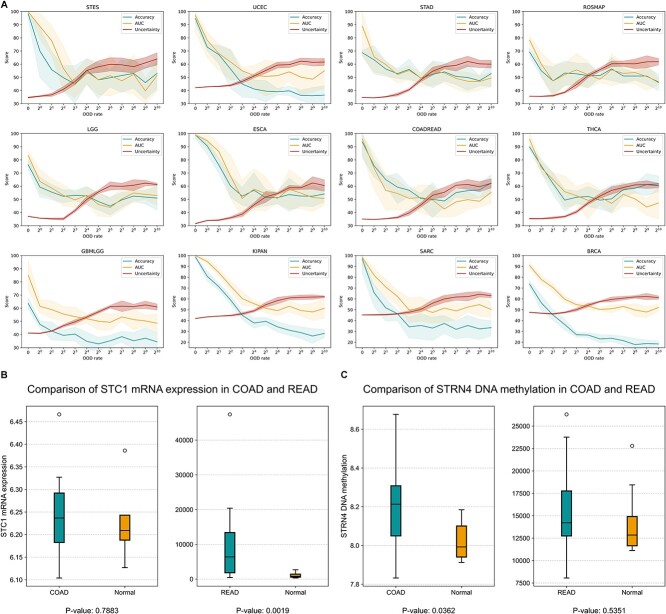
Uncertainty analysis and biomarker identification. (**a**) Dirichlet uncertainty analysis of multiomics data with different levels of noise. To validate the effectiveness of the uncertainty metrics, we conducted an experiment where we added Gaussian noise with varying degrees to each dataset and observed the classification performance of HTML. The results are plotted on a graph, where three lines represent accuracy, AUROC (AUC), and uncertainty respectively. To ensure the reliability of our results, we calculated the 95% confidence interval of the results based on 5-fold cross-validation and represented it with shaded areas. The results showed that as the noise level in the data increased, the classification performance of the model continued to decline, accompanied by a continuous increase in the model’s uncertainty. This trend was observed in all 12 datasets. (**b**) We investigated the expression difference of STC1 between normal and COAD as well as that between normal and READ. Expression alteration is absent in normal and COAD samples but exists in normal and READ samples (*P* value = 0.0019). (**c**) We investigated the expression differences in STRN4 between normal and COAD tissues as well as between normal and READ tissues. Expression alteration is absent in normal and READ samples but exists in normal and COAD samples (*P* value = 0.0363).

To validate the efficacy of the uncertainty metrics, we visualized the changes in the performance of HTML using the in−/out-of-distribution (ID/OOD) samples. Here, we set the original samples as ID and the samples with added Gaussian noise as OOD. Specifically, we added Gaussian noise with varying standard deviations (i.e. $s={2}^k,k=0,1,2,.\dots, 10$) to the test samples. We conducted 5-fold cross-validation on all ID and OOD samples for each dataset in the 12 datasets and calculated the mean value of classification accuracy, AUROC, and uncertainty score of HTML for each dataset under each distribution setting as well as their 95% confidence intervals, as presented in Figure 4. The information in Figure 4 effectively confirms the usefulness and validity of the uncertainty metrics. In all datasets, in-distribution data gain the highest classification performance and lowest uncertainty score. As the data distribution deviates to a greater extent, the classification performance of the model gradually deteriorates, while the uncertainty scores of the model’s classification results continue to increase. We can infer that in situations where there is a significant amount of data noise, the model’s ability to accurately and consistently predict and classify may be compromised. As such, the uncertainty metric can serve as a reliable compass in evaluating the credibility of the data distribution.

### HTML identifies important biomarkers

Our research on subtype classification has led to the identification of a variety of DNA methylation, mRNA, and miRNA biomarkers that warrant further investigation for their potential role in the diagnosis and treatment of distinct cancer subtypes. A selection of noteworthy biomarkers is presented in Supplementary Tables 8–19 available online at http://bib.oxfordjournals.org/ and Table 2. To showcase the value of our findings, we chose certain biomarkers from the COADREAD dataset (comprising COAD and READ cancer subtypes) as examples.

**Table 2:** Important biomarkers identified by HTML. We listed the important biomarkers found by HTML for further investigation of each cancer subtype in 10 datasets. These biomarkers will serve as potential targets for future research and may lead to novel diagnostic or therapeutic approaches for cancer treatment

COADREAD	COAD	meth	LRMP, TCF4, OXTR, FLJ39609, UGT2B10
		mRNA	TNKS1BP1, BTBD6, TMEM129, STRN4, DGKQ
		miRNA	hsa-mir-411, hsa-mir-101-2, hsa-mir-1185-1, hsa-mir-135a-1, hsa-mir-134
	READ	meth	DDIT4L, ZNF461, FSTL5, NRAP, GPR150
		mRNA	ZFP36L2, SLC25A39, SLC37A1, PLAUR, SPON1
		miRNA	hsa-mir-765, hsa-mir-541, hsa-mir-1295, hsa-mir-514-1, hsa-mir-181c
ESCA	ESCC	meth	SPRR1B, GIPC2, KRT73, ETNK1, GZMH
		mRNA	CDCA4, SENP5, C10orf58, USP5, FXR1
		miRNA	hsa-mir-1294, hsa-let-7a-3, hsa-mir-200c, hsa-mir-10b, hsa-mir-184
	EAC	meth	ME3, RDH5, BCL2L15, CLCA3P, IL18
		mRNA	GPR87, PFN2, STON2, PL-5283, MLF1
		miRNA	hsa-mir-24-1, hsa-mir-339, hsa-mir-151, hsa-mir-205, hsa-mir-3605
GBMLGG	AST	meth	PHLDA2, ARHGEF5L, IMPACT, LOC113230
		mRNA	LPPR1, BAP1, ANAPC5, ZNF33B, VAPA
		miRNA	hsa-mir-380, hsa-mir-548o, hsa-mir-3199-1, hsa-mir-204, hsa-mir-1301
	ODG	meth	PTENP1, SLMO1
		mRNA	SLC24A3, HMP19, GNAI1, SF1, RNASEH2C
		miRNA	hsa-mir-3909, hsa-mir-383, hsa-mir-3662, hsa-mir-3200, hsa-mir-2116
	OAC	meth	MLH3, CYP51A1, GPR19, HSPA1A
		mRNA	PTCD3, ECE2, LETMD1, FXR2, PRPF6
		miRNA	hsa-mir-338, hsa-mir-22
SARC	LMS	meth	TNFSF4, NPB, COL12A1, SNX22, SLCO2A1
		mRNA	PLP2, LSM7, U2AF1, DLGAP4, ADRBK1
		miRNA	hsa-mir-1228, hsa-mir-196a-1, hsa-mir-744, hsa-mir-551b, hsa-mir-664
	DDLPS	meth	GPR19, TRIM58, SNORD17, LOC391322
		mRNA	MCTS1, NPLOC4, MED16, RNF167, FAM125A
		miRNA	hsa-mir-195, hsa-mir-215, hsa-mir-1292, hsa-mir-548 k, hsa-mir-409
	others	meth	FAM115A, CYR61, FLJ13224, SEPT10, MTAP
		mRNA	WHSC1, C1orf151, PRPF19, HMGN2, THOC5
		miRNA	hsa-mir-668, hsa-mir-363, hsa-mir-720, hsa-mir-590, hsa-mir-133a-2
STAD	`ADC	meth	RPL39L, HIST1H3J, KIT, LOC80154, NSUN7
		mRNA	C1orf144, RHOA, TOMM20, ARPP19, IER5L
		miRNA	hsa-mir-328, hsa-let-7f-2, hsa-mir-374b, hsa-mir-149, hsa-mir-3130-1
	IAC	meth	NCRNA00152, MDS2, CCDC15, B3GALT4, NTN3
		mRNA	RACGAP1, CTBP2, YIPF3, RRM2B, C9orf152
		miRNA	hsa-mir-489, hsa-mir-551a, hsa-mir-378, hsa-mir-582, hsa-mir-34a
STES	ESCC	meth	MIR192, ETNK1, LOC80054, KRT71, FZD5
		mRNA	MARK4, GLI3, KRI1, TMEM189, ABCF3
		miRNA	hsa-mir-1292, hsa-mir-30c-2, hsa-mir-128-1, hsa-mir-103-1
	EAC	meth	LCE2D, POSTN, GFM1, FAM153A, CFTR
		mRNA	DGKA, A4GALT, ALS2CR4, DFNA5, DRAP1
		miRNA	hsa-mir-192, hsa-mir-3942, hsa-mir-125b-1
THCA	Usual type	meth	DNAH1, SLC23A1, TUBB6, KLHDC5, OR6Q1
		mRNA	APOC1, FAM171B, CCDC149, MPPE1
		miRNA	hsa-mir-526b, hsa-mir-3117, hsa-mir-433, hsa-mir-16-2, hsa-mir-132
	Unusual type	meth	TAGLN, TRIP4, PTGIS, NCRNA00152, LTF
		mRNA	WSB2, TMEM80, KIAA1191, RPN1
		miRNA	hsa-mir-449b, hsa-mir-186, hsa-mir-148a, hsa-mir-3926-1, hsa-mir-190b
UCEC	EEA	meth	DMC1, MIR7–1, PRAMEF21, MIR138–1, TREX1
		mRNA	ZNF512
		miRNA	hsa-mir-485, hsa-mir-1266, hsa-mir-16-1, hsa-mir-3926-1, hsa-mir-15b
	SEA	meth	UBLCP1, KRTAP5–6, TEC
		mRNA	
		miRNA	hsa-mir-3191, hsa-mir-3944, hsa-mir-346, hsa-mir-616, hsa-mir-138-1
	MSEAC	meth	SLC18A1, KLHDC7A, FAM167A, ARID1A, LOC400931
		mRNA	RPS18, EPCAM, PAX8, DHX36, HDAC5
		miRNA	hsa-mir-1910, hsa-mir-3913-1, hsa-mir-27b, hsa-mir-143, hsa-mir-431
BRCA	Normal-like	meth	CXCL1, COL11A2, RAET1L
		mRNA	ALDH1A3, ZNF80, SLC30A10
		miRNA	hsa-mir-3676, hsa-mir-489, hsa-mir-320b-2
	Basal-like	meth	MAPK4, ABCC11, TRIML2, LEMD1, GJB3
		mRNA	MIR190B, VLDLR
		miRNA	hsa-mir-3194
	HER2-enriched	meth	PI3, C16orf57, MIA, MGC3771
		mRNA	TMEM22, ZSWIM2
		miRNA	hsa-mir-26a-1, hsa-mir-144, hsa-mir-19b-1, hsa-mir-199a-1
	Luminal B	meth	RGS20
		mRNA	C15orf62, HAS2AS
		miRNA	hsa-mir-1266
KIPAN	KICH	meth	FOXL1, GJC2, C5orf4, COMT, NRG4
		mRNA	CDH1, CTDSPL, CCL2, FOXI2, ASTN2
		miRNA	hsa-mir-1291, hsa-mir-660, hsa-mir-548 s, hsa-mir-363, hsa-mir-3934
	KIRC	meth	LGALS9B
		mRNA	KLHL3, RPL28, CLU, INSR, THY1
		miRNA	hsa-mir-342, hsa-mir-146b, hsa-mir-514-2, hsa-mir-495, hsa-mir-320d-2
	KIRP	meth	IL18BP, RPS6
		mRNA	ANTXR1, GPI,FKBP10, COBLL1, TACC1
		miRNA	hsa-mir-153-2, hsa-mir-1229, hsa-mir-30c-1, hsa-mir-3944, hsa-mir-148a
LGG	Grade 2	meth	TPPP3, ITPRIPL1, PSG1
		mRNA	TMEM30A, RTN3, HNRNPH1, STMN3, PDLIM5
		miRNA	hsa-mir-218-1, hsa-mir-3609, hsa-mir-3614, hsa-mir-378c, hsa-mir-27a
	Grade 3	meth	UGT2B28, DNASE1L3, ELSPBP1
		mRNA	OSGIN2, CASC4, ATPAF1, STIP1, LOC643763
		miRNA	hsa-mir-448, hsa-mir-3200, hsa-mir-370, hsa-mir-627, hsa-mir-200b

Within the COAD biomarkers, LRMP has been identified as hypermethylated and downregulated in a subpopulation of highly mobile cells, known as MG cells, derived from a COAD cell line. These cells are distinguished by their migratory capacity and epithelial-mesenchymal transition attributes, which are indicative of the metastatic potential and malignancy of COAD. Tanaka *et al.* [44] conducted a study that demonstrated the significant impairment of MG cell migration with the demethylation and upregulation of LRMP and other genes, suggesting a correlation between LRMP methylation and COAD. This relationship is further supported by the findings of various other studies, including the identification of COAD marker genes through single-cell transcriptomic analysis [45] and gene expression analysis of COAD and normal samples via RT-PCR assay [46]. In addition, genes identified in mRNA expression features, such as TNKS1BP1 [47], which encodes a protein involved in telomere replication, and STRN4 [48], which encodes a scaffolding protein with multiple functions, have also been reported to play a role in COAD tumorigenesis. To further investigate this, we analysed the expression differences of STRN4 between normal and COAD samples using data from Snipstad *et al.* [49] and compared them to those between normal and READ samples using data from Zuurbier *et al.* [50], as shown in Supplementary Table 20 available online at http://bib.oxfordjournals.org/ and Figure 4b. We discovered a consistent result, with a significant expression alteration present in normal and COAD samples (*P* value = 0.03565) but absent in normal and READ samples. In terms of miRNA expression features, hsa-mir-101-2 was identified as one of the prognostic miRNA signatures for COAD in a study by Lv *et al.* [51]. The targets of this signature are predicted to enrich biological process terms, such as focal adhesion and transcription disorders in cancer.

In terms of biomarkers for READ, STC1 is a member of the secretory glycoprotein family and is involved in processes such as apoptosis and inflammation. Its association with READ has been reported [52], and its secretion by tumor stromal cells may contribute to READ metastasis by mediating PDGF receptor signaling [53]. We analysed the same dataset mentioned earlier to examine the differential expression of STC1 in normal and COAD samples or normal and READ samples, and the results are shown in Supplementary Table 21 available online at http://bib.oxfordjournals.org/ and Figure 4c. We found a significant difference in STC1 expression between normal and READ with a *P* value close to 0.001, which suggests that STC1 could be a biomarker for READ. Among the genes identified in the mRNA expression features, SPON1 is a coding gene that produces a product predicted to be secreted to the extracellular matrix and is involved in cell adhesion, a process that could be dysregulated in tumorigenesis. Supiot *et al.* [54] examined its expression before and after preoperative radiotherapy in READ patients and found a significant upregulation. Similarly, upregulation of hsa-mir-765 was found to occur in READ patients in response to neoadjuvant chemoradiotherapy [55]. These findings suggest that SPON1 and hsa-mir-765 could be potential biomarkers for READ. Moreover, TargetScan predicted possible target genes of hsa-mir-765, including PDX1 [56], KLK4 [57] and LHPP [58], all of which have been reported to correlate with READ.

## DISCUSSION

In this study, we proposed a highly trustworthy multiomics learning framework (HTML), for personalized pancancer and cancer subtype diagnosis and prognosis. Through training on pancancer and cancer subtype datasets, HTML has the potential to seamlessly integrate multiple modalities of data, thereby enabling personalized medical diagnosis. This innovative technology is poised to overcome the existing barriers between pancancer diagnosis and cancer subtype determination, thereby achieving a comprehensive diagnosis pipeline. This pipeline is capable of processing one or more pieces of omics information from patients. It initially predicts the presence of cancer and the specific type of cancer and subsequently identifies the specific subtype of the patient’s cancer, which has the potential to integrate the entire diagnostic process into one model.

Moreover, conventional models are unable to conduct sample adaptive analysis based on the unique data distribution characteristics of each sample and lack the ability to validate and verify the reliability of their own classification results. To address this challenge, we developed the HTML model, which leverages dynamic learning at both the feature and modality levels and Dirichlet uncertainty learning. This innovative model efficaciously integrates information across diverse modalities of data, enabling it to make cutting-edge classification predictions with remarkable accuracy, enabling the precise identification and diagnosis of cancer-causing genes for individuals, thereby making personalized treatment possible.

HTML’s feature dynamic weight mechanism assigns a unique weight to each feature. This innovative approach enables us to identify potential biomarkers that can differentiate between various types of cancers and cancer subtypes, thereby laying the groundwork for further exploration of the biological mechanisms that underpin cancer development.

Despite utilizing only DNA methylation, mRNA expression and miRNA expression data for our multiomics classification tasks, our HTML framework boasts impressive extensibility and can be customized to accommodate various types of data (such as SNVs, copy number or even medical images and texts) by adjusting the model parameters accordingly. In this way, HTML represents a highly adaptable supervised multiomics classification framework with exceptional interpretability and extensibility.

## METHODS

### Feature dynamic learning

The multiomics input can be formulated as ${\mathbf{V}}_i\in{\mathbb{R}}^{n\times{k}_i},0<i\le n$. Here, ${\mathbf{V}}_1,{\mathbf{V}}_2,\dots, {\mathbf{V}}_m$ represent $m$ distinct modalities, with each modality containing $n$${k}_i$-dimensional vectors. The quantity $n$ denotes the number of samples, and the feature volume ${k}_i$ varies across different modalities. The symbol $\mathbb{R}$ represents the set of real numbers.

Suppose we have a single omic data input ${\mathbf{V}}_i=\left\{{S}_1,{S}_2,\dots, {S}_m\right\},S\in{\mathbb{R}}^{k_i}$, where each sample $S$ is represented by a ${k}_i$-dimensional vector $S=\left[{s}_1,{s}_2,\dots, {s}_{k_i}\right]$. Our objective is to learn a feature dynamic module that generates a dynamic weight vector $W=\left[{w}_1,{w}_2,\dots{w}_{k_i}\right]\in{\mathbb{R}}^{k_i}$ that performs self-adaptive feature selection. We can achieve this by defining a transformation with the input feature to obtain the dynamic feature embedding ${S}_d$ as follows:


$$ {S}_d=\text{Tanh}\left(W\cdotp \sigma (S)\right)=\text{Tanh}\left(\left[\frac{w_1}{1+{e}^{-{s}_1}},\frac{w_2}{1+{e}^{-{s}_2}},\dots, \frac{w_{k_i}}{1+{e}^{-{s}_{k_i}}}\right]\right)\!\!\Bigg) $$

where $W$ is generated through an MLP network and has the same dimension as the input $S$, $\sigma$ represents the sigmoid function, and the sigmoid and hyperbolic tangent functions here aim to introduce nonlinear parameters in the learning process.

The concept of feature dynamics can effectively address the issue of overfitting, in which the model tends to fit the noise in the input data rather than the underlying patterns [59]. Through the adaptive selection of features based on their significance to the prediction task, the model can improve its performance when encountering new samples and can achieve a more meaningful interpretation of the input features.

### Methylation-guided attention

Through the feature selection module, we obtained a better interomics feature representation ${S}_d$ for each sample. Considering the uniqueness of multiomics data and their biological meaning, DNA methylation levels largely affect the expression levels of mRNA and miRNA. Therefore, we proposed a DNA methylation-guided attention mechanism to model the interomics relationships among different omics data.

In HTML, the original DNA methylation input features serve as the query vectors $d=\left[{d}_1,{d}_2,\dots, {d}_k\right]$, while the mRNA or miRNA input features $m=\left[{m}_1,{m}_2,\dots, {m}_k\right]$ act as key and value vectors. Both $d$ and $m$ have been projected into the same vector space. The guided attention context vector ${S}_a=\left[{c}_1,{c}_2,\dots, {c}_k\right]$ is then calculated as:


$$ {c}_i=\sum_{j=1}^k{a}_{ij}{m}_j $$

where ${a}_{ij}$ represents the attention weight between ${d}_i$ and ${m}_j$ and $k$ represents the feature numbers of both vectors. The mRNA context vector ${c}_i$ is computed by the sum of all attention values on DNA methylation features as follows:


$$ {a}_{ij}=\frac{\text{score}\left({d}_i,{m}_j\right)}{\sum_{j^{\prime }=1}^k\text{score}\left({d}_i,{m}_{j\prime}\right)} $$

where the alignment $\text{score}$ function indicates how well the elements of the DNA features align with the mRNA features at the position and the weights ${a}_{ij}$ are computed by applying a Softmax operation to the previously computed alignment scores as follows:


$$ \text{score}\left({d}_i,{m}_j\right)=\frac{d_i^{\top }{W}_a{m}_j}{\sqrt{k}} $$

where $\sqrt{k}$ is the scaling factor and ${W}_a$ is the multiplicative attention weight matrix.

After the feature dynamic module and methylation-guided attention, we obtained both the inner-omics feature representation ${S}_d$ and interomics feature representation ${S}_a$. We then took the average of vectors ${S}_d$ and ${S}_a$ ($S$ for DNA methylation) to obtain the final representation vector $\hat{S}=\frac{1}{2}\left({S}_d+{S}_a\right)$ for each omics.

### Triple contrastive learning

To address the interactions among multiomics data and improve the classification task accuracy, we proposed a triple contrastive loss (TCL) function for learning comparisons among multiple modalities. This loss function brings together the distances among the representations ${x}_1,{x}_2$ and ${x}_3$ of all modalities ($3$ in our experiment) of a given sample while pulling away the representations ${x}_j$ of other samples:


\begin{align*} &{L}_{\text{TCL}}\\ & \!=-\frac{1}{m}\sum_{i=1}^m\log \frac{\sum_{i=1}^3\exp \left({\mathbf{x}}_i^{\top }{\mathbf{x}}_{\left(i \operatorname{mod}\ 3\right)+1}/\tau \right)}{\sum_{i=1}^3\!\exp \left({\mathbf{x}}_i^{\top }{\mathbf{x}}_{\left(i \operatorname{mod}\ 3\right)+1}/\tau \right)\!+\!\sum_{j=1}^{3K}\!\exp \left({\mathbf{x}}_{\left(j \operatorname{mod}\ 3\right)}^{\top }{\mathbf{x}}_j/\tau \!\right)} \end{align*}

where $m$ is the number of samples, $\tau$ is a temperature parameter, ${x}_{3i+1}$ is the representation of the $i$-th DNA methylation feature, ${x}_{3i+2}$ is the representation of the $i$-th mRNA expression feature, ${x}_{3i+3}$ is the representation of the $i$-th miRNA expression feature, and $K$ is the number of negative samples. This process optimizes the representation vectors of each modality and improves the accuracy of the overall task.

### Modality dynamic learning

As with feature dynamic learning, the contribution of individual features varies in each modality toward the final classification outcome. This holds true for disparate modalities as well. To effectively account for each modality’s contribution to the final classification, HTML introduces a modality dynamic learning module to compute the model’s confidence (MCC) for different modalities. Take the DNA methylation feature $\hat{S}=\left[{\hat{d}}_1,{\hat{d}}_2,\dots, {\hat{d}}_k\right]$ as an example:


$$\text{MCC}\left(\hat{S}\right)=\sigma \left(\hat{S}\cdotp{W}_{\text{methyl}}\right)=\frac{1}{1+\exp \left(-\sum_{i=0}^k{\hat{d}}_i\cdotp{w}_i\right)}$$

where ${W}_{\text{methyl}}=\left[{w}_1,{w}_2,\dots, {w}_k\right]\in{\mathbb{R}}^k$ is the weight parameter for DNA methylation features of the modality dynamic learning module, and each MCC function returns a confidence score for each modality feature of each sample.

### Dirichlet uncertainty learning

The Dirichlet distribution is a prevalent multivariate probability model used for random variable synthesis [60]. It is expressed in terms of $k$ concentration parameters, denoted $\alpha =\left[{\alpha}_1,{\alpha}_2,\dots, {\alpha}_k\right]$. The probability density function of the Dirichlet distribution is given by:


$$\text{Dir}\left(\boldsymbol{\mu} \mid \boldsymbol{\alpha} \right)=\left\{\begin{array}{ll}\frac{1}{B\left(\boldsymbol{\alpha} \right)}\prod_{i=1}^K{\mu}_i^{\alpha_i-1}& \text{for}\ \boldsymbol{\mu} \in{U}_k,\\{}0& \text{otherwise}\end{array}\right.$$

where $B\left(\boldsymbol{\alpha} \right)$ represents the beta distribution parameterized by $\boldsymbol{\alpha}$, and ${\mathcal{U}}_k$ denotes the $k-1$ dimensional unit simplex, defined as:


$${U}_k=\left\{\boldsymbol{\mu} \mid \sum_{i=1}^k{\mu}_i=1\ \text{and}\ 0\le{\mu}_1,\dots, {\mu}_K\le 1\right\}$$

It is noteworthy that each element in $\boldsymbol{\alpha}$ corresponds to a different category concentration, and $\boldsymbol{\alpha}$ is $K$-dimensional. From the Dirichlet distribution equation definition, we inferred that each unit of the vector representing the category concentration can parameterize a corresponding Dirichlet distribution. By associating the $\boldsymbol{\alpha}$ vector with the input class probabilities, one obtains a full perspective of the overall distribution of class probability beyond simply relying on the highest classification probability.

We introduce the Dempster–Shafer Theory of Evidence (DST) [61], which utilizes belief mass to allocate subjective probabilities in describing an opinion’s credibility in order to assign an uncertainty score for each modality [19]. A belief mass indicates how reliable the predicted label is, while the overall uncertainty denotes how much the total probability of the class should be questioned. The belief mass and overall uncertainty can be quantified through:


$$ \left\{\begin{array}{l}{b}_k=\frac{e_k}{S}\\{}u=\frac{K}{S}\end{array}\right.S=\sum_{k=1}^K\left({e}_k+1\right) $$

where $K$ represents the number of classification types, and ${e}_k$ indicates supporting evidence for the belief mass. In the case of multitype classification, the evidence ${e}_k$ is supported by the output from the neural network. The belief mass function and the overall uncertainty are related as follows: 


$$ {u}^m+\sum_{k=1}^K{b}_k=1 $$

This supports the notion that the belief mass and overall uncertainty interact complementarily. In our experiments, the Dirichlet distribution allowed modelling of secondary probability and uncertainty beyond relying solely on the highest probability as the selected class [43]. Utilizing the probabilistic output ${e}_k$ as evidence from the neural network, we defined the Dirichlet distribution parameters ${\boldsymbol{\alpha}}_k={e}_k+1$ to ensure that the value of $\boldsymbol{\alpha}$ satisfies the mathematical requisite that $\boldsymbol{\alpha} -1$ in the Dirichlet distribution must be nonnegative.

### Dempster–Shafer multiomics integration

Once the determination of the belief mass, overall uncertainty and evidence is complete, a quantitative measure of the reliability of opinions can be obtained. Nevertheless, in multiple modality tasks, integrating the belief mass and overall uncertainty from various sources proves to be a challenging task. The integration rule should consider the uncertainty value instead of simply reconciling the belief mass values from different sources. Additionally, multimodal integration must account for conflicting opinions across diverse modalities. Given these requirements, we proposed the following fusion rule. The integrated uncertainty value is calculated as follows: 


$$ u=1/\left(1-C\right){u}^1{u}^2,C=\sum \limits_{i\ne j}{b}_i^1{b}_j^2 $$

The variable $C$ represents a measure proposed to assess the conflicting results of different opinions. In cases where opinions are at odds with each other, the sum of the belief mass product is calculated. As the frequency of conflicting scenarios increases, the value of $C$ rises, resulting in an increase in the integrated uncertainty value, leading to a less persuasive integrated result. Concerning the fused belief mass, we set the integration rule as follows:


$$ {b}_k=\frac{1}{1-C}\left({b}_k^1{b}_k^2+{b}_k^1{u}^2+{b}_k^2{u}^1\right) $$

The cross-relation between the belief mass and the overall uncertainty distributes different weights for distinct modalities linked to their respective uncertainties. For instance, when the uncertainty value of modality 1 is higher than that of modality 2, the integrated ${b}_k$ may rely more heavily on the input of ${b}_2$ thanks to the introduction of ${b}_k^2{u}^1$, and vice versa. The DST integration rule can be easily extended to multiomics scenarios where the number of omics exceeds 2. If we use M to denote both the belief mass and the overall uncertainty: $ \textsf{M}=\left\{{\left\{{b}_k\right\}}_{k=1}^K,u\right\} $, we can summarize the integration process between two modalities $ {\textsf{M}}^1 $ and $ {\textsf{M}}^2 $ as: 


$$ \textsf{M}={\textsf{M}}^1\oplus{\textsf{M}}^2 $$

where the operator $\oplus$ represents the integration process. This integration rule is suitable for commutativity and associativity. The fusion result is independent of the order of fusion and can effectively be applied to more than two modalities as follows: 


\begin{align*} \textsf{M}={\textsf{M}}^1\oplus{\textsf{M}}^2\oplus \cdots{\textsf{M}}^m \end{align*}

The loss function for DST integration methods comprises the cross-entropy loss in each modality: 


$${L}_{\text{single}}=\sum_{m=1}^M\left(-\sum_{k=1}^K{y}^m\log \left({b}_k^m\right)\right)$$

where $M$ is the number of modalities, and each ${b}_k$ represents the predicted probability. The loss function for the final classification outcome contains a cross-entropy loss and a KL divergence loss


$${L}_{\text{integrate}}=-\sum_{i=1}^c{\text{label}}_i\left(\psi \left(\sum_{j=1}^c{\alpha}_j\right)-\psi \left({\alpha}_i\right)\right)-\text{KL}\left(\alpha, \gamma \right)$$

where


\begin{align*} \text{KL}\left(\alpha, \gamma \right) =\ & {\text{D}}_{\text{KL}}\left[\text{Dir}\left({\boldsymbol{\mu}}^m\mid{\overset{\sim }{\boldsymbol{\alpha}}}^m\right)\parallel \text{Dir}\left({\boldsymbol{\mu}}^m\mid{\overset{\sim }{\boldsymbol{\gamma}}}^m\right)\right]\\{} =\ & \log \left(\frac{\varGamma \left(\sum_{k=1}^K{\overset{\sim }{\alpha}}_k\right)}{\varGamma (K)\prod_{k=1}^K\varGamma \left({\overset{\sim }{\alpha}}_k\right)}\right)\\{}& +\sum_{k=1}^K\left({\overset{\sim }{\alpha}}_k-1\right)\left[\psi \left({\overset{\sim }{\alpha}}_k\right)-\psi \left(\sum_{j=1}^K{\overset{\sim }{\alpha}}_j\right)\right]\end{align*}

where $\varGamma \left(\cdotp \right)$ is the gamma function and ${\overset{\sim }{\boldsymbol{\alpha}}}^m=\mathbf{y}+\left(1-\mathbf{y}\right)\odot{\boldsymbol{\alpha}}^m$ is the Dirichlet distribution after replacing the ${\alpha}_k$ corresponding to the ground truth label with 1, thus avoiding penalizing the Dirichlet parameter of the ground truth class to 1, and ${\overset{\sim }{\boldsymbol{\alpha}}}^m$ is the unit vector of length $k$.

We formulated our objective function for HTML by combining the triple contrastive loss, individual cross-entropy loss, and integrated loss. To further enhance the classification results, we incorporated uncertainty metrics and an ${\mathcal{L}}_1$ regularization term to mitigate overfitting. This comprehensive objective function aims to reduce uncertainty and improve the accuracy of the classification results:


$${L}_{\text{overall}}={L}_{\text{TCL}}+{L}_{\text{single}}+{L}_{\text{integrate}}+u+0.0001\ast \parallel w{\parallel}_1$$

**Key Points**

- We present a novel and highly personalized approach to cancer diagnosis and prognosis
- Our method outperforms static-architecture-based methods in various clinical tasks.
- Our method showcases the effectiveness of individualized treatment with a strong emphasis on explainability
- Our method incorporates uncertainty metrics to provide reliable indications of the model’s output
- Our method contributes to advancing biological research by identifying potential biomarkers

## Supplementary Material

Supplementary_Information_bbad378

## Data Availability

The BRCA and ROSMAP datasets were obtained from the GitHub repository of MOGONET (https://github.com/txWang/MOGONET). The omics data of the other datasets (COADREAD, ESCA, GBMLGG, SARC, STAD, STES, THCA, UCEC, LGG, KIPAN), as well as the survival time information, were obtained from The Cancer Genome Atlas (TCGA) Program through Broad GDAC Firehose (https://gdac.broadinstitute.org/). The PAM50 breast cancer subtypes of TCGA BRCA patients were obtained through the TCGAbiolinks R package (v2.12.6, http://bioconductor.org/packages/release/bioc/html/TCGAbiolinks.html). We have made our preprocessed data for the 12 cancer subtype dataset available through Google Drive (https://drive.google.com/drive/folders/1_tJ2ekhTmWp7ZcRVjUVGx0cqGMRKEhNo?usp=share_link).

## CODE AVAILABILITY

The source codes, trained models, demos and tutorials for this work can be found at GitHub (https://github.com/YuxingLu613/HTML).

## Author Biographies

**Yuxing Lu** is currently a PhD student at the Department of Big Data and Biomedical AI, College of Future Technology, Peking University, Beijing, China. His research primarily focuses on multi-omics data integration and metabolomic knowledge graphs.

**Rui Peng** is currently a PhD student in the Academy for Advanced Interdisciplinary Studies, Peking University. His research interests focus on multi-omics integration and computational biology.

**Lingkai Dong** is a masters student in chemical and biomolecular engineering at The Hong Kong University of Science and Technology. His research interests include drug delivery and cancer targeting therapy.

**Kun Xia** is a master student in School of Software and Microelectronics, Peking University.

**Renjie Wu** is a bachelor student in School of Life Science, Peking University.

**Shuai Xu** is currently a PhD student at the Institute of Molecular Medicine, College of Future Technology, Peking University, Beijing, China. His research primarily focuses on the comprehensive analysis of multi-omics data obtained from single-cell sequencing.

**Jinzhuo Wang** is a research assistant professor in the Department of Big Data and Biomedical AI, College of Future Technology, Peking University. His research interest is multimodal biomedical AI.
