# Supplementary material for: Multiomics dynamic learning enables personalized diagnosis and prognosis for pancancer and cancer subtypes
Source: Brief Bioinform. 2023 Oct 26;24(6):bbad378. doi: 10.1093/bib/bbad378 (PMC10605059; doi:10.1093/bib/bbad378)
Supplement: Supplementary_Information_bbad378 [file supplementary_information_bbad378.pdf]

Supplementary Information: Multiomics dynamic learning enables personalized diagnosis and prognosis for pan-cancer and cancer-subtypes

Contents

1 Supplementary Figures **3**

Figure S1: Overall pipeline of HTML 3

Figure S2: Performance in the pan-cancer dataset 3

2 Supplementary Tables **4**

Table S1: Overview of pan-cancer dataset 4

Table S2: Overview of cancer sub-type datasets 4

Table S3: Performance comparison on the pan-cancer dataset 5

Table S4: Ablation study of HTML 6

Table S5: Hyperparameter selection of HTML 6

Table S6: Result comparison under different combination settings . . 6

Table S7: Abbreviation explanation 7

Table S8: Biomarkers in COADREAD dataset 8

Table S9: Biomarkers in ESAC dataset 9

Table S10: Biomarkers in GBMLGG dataset 10

Table S11: Biomarkers in SARC dataset 11

Table S12: Biomarkers in STAD dataset 12

Table S13: Biomarkers in STES dataset 13

Table S14: Biomarkers in THCA dataset 14

Table S15: Biomarkers in UCEC dataset 15

Table S16: Biomarkers in BRCA dataset 15

Table S17: Biomarkers in KIPAN dataset 16

Table S18: Biomarkers in LGG dataset 17

Table S19: Biomarkers in ROSMAP dataset 18

Table S20: Comparison of STC1 mRNA expression 19

Table S21: Comparison of STRN4 DNA methylation 20

3 Supplementary Discussions **21**
Data overview 21
Data preprocessing 21
Training Details 22
Biomarker identification with HTML 22
Biomarkers diverge in individuals and cancer types 23
Comparison with statistical methods 23

Reference **24**

1 Supplementary Figures

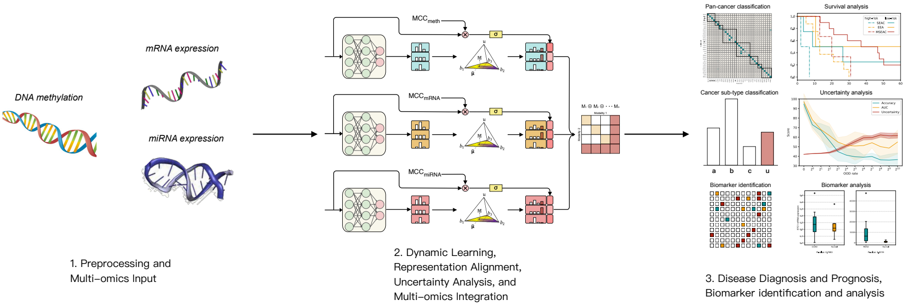

Fig. 1 Overall pipeline of HTML. HTML framework takes multi-omics data, including DNA methylation, mRNA expression, and miRNA expression, as inputs. It employs various levels of dynamic learning, several modality alignment methods, and an uncertainty integration method to perform multi-omics integrated learning. HTML framework has demonstrated its capability in numerous downstream tasks, such as pan-cancer classification, sub-cancer identification, survival prediction, uncertainty prediction, biomarker identification, and analysis.

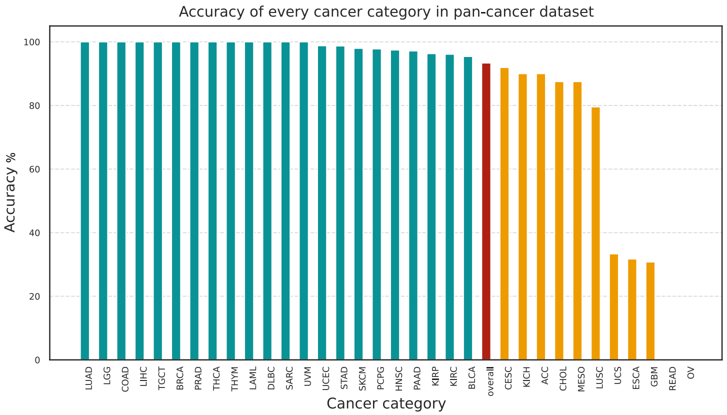

Fig. 2 Classification performance of each cancer category in the pan-cancer dataset. We conduct a statistical analysis on the classification results of HTML models for each cancer category in the pan-cancer dataset, and find that the classification accuracy of most cancer categories is above 95%. Only a few categories, such as USC, ESCA, GBM, READ, and OV, have lower classification accuracy, which may be due to 1. high similarity between these cancer types and certain other cancer types, making the model easily confused; 2. particularly small sample sizes for these cancer types, making it difficult for the model to learn effectively.

2 Supplementary Tables

Table 1 Overview of pan-cancer classification dataset. We present the statistics of the pan-cancer dataset, which includes 33 different cancer types. Specifically, we calculate the number of samples in each cancer type and generated a table that shows the number of features before and after the preprocessing step.

	Features before preprocessing (DNA meth, mRNA)			485,577, 60,666	Features after preprocessing (DNA meth, mRNA)			10,176, 15,165
Label	BRCA	THCA	PRAD	LGG	HNSC	LUAD	SKCM	UCEC
Count	869	562	536	528	524	489	473	457
Label	BLCA	LIHC	LUSC	KIRC	STAD	COAD	CESC	KIRP
Count	426	414	377	345	338	322	309	298
Label	SARC	PCPG	PAAD	ESCA	TGCT	LAML	THYM	READ
Count	263	187	183	172	139	133	122	99
Label	MESO	UVM	ACC	KICH	GBM	UCS	DLBC	CHOL
Count	87	80	79	65	62	57	48	44
Label	OV							
Count	7							

Table 2 Overview of cancer sub-type classification datasets. In our experiment, we use 12 different cancer sub-type classification datasets and report the name of the cancer sub-type labels present in each dataset, as well as the number of features before and after the preprocessing step.

Dataset	Samples	Labels	Features before preprocessing (DNA meth, mRNA, miRNA)	Features after preprocessing (DNA meth, mRNA, miRNA)
COADREAD	332	COAD: 254, READ: 78	20,113, 20531, 420	2000, 2000, 420
ESCA	193	ESCC: 94, EAC: 89	20,101, 20531, 520	2000, 2000, 520
GBMLGG	511	AST: 193, ODG: 191, OAC: 127	20,114, 20,531, 548	2000, 2000, 548
SARC	257	LMS: 103, DDLPS: 58, others: 96	20,097, 20,531, 508	2000, 2000, 508
STAD	371	ADC: 205, IAC: 166	20,101, 20,531, 507	2000, 2000, 507
STES	193	ESCC: 94, EAC: 89	20,100, 20,531, 511	2000, 2000, 511
THCA	500	Usual type: 356, Unusual type: 144	20, 118, 20,531, 517	2000, 2000, 517
UCEC	430	EEA: 311, SEA: 98, MSEAC: 21	20, 118, 20,531, 554	2000, 2000, 554
BRCA	875	Normal-like: 115, Basal-like: 131, HER2-enriched: 46, Luminal A: 436, Luminal B: 147	20,531, 20,106, 503	1000, 1000, 503
LGG	510	Grade 2: 246, Grade 3: 264	20,531, 20,114, 548	2000, 2000, 548
KIPAN	631	KICH: 66, KIRC: 318, KIRP: 274	20,531, 20,111, 445	2000, 2000, 445
ROSMAP	351	NC: 169, AD: 182	55,889, 23,788, 309	200, 200, 200

Table 3 HTML's performance comparison on the pan-cancer classification tasks. We compare the performance of HTML with other state-of-the-art methods and observe that HTML outperforme them in all evaluation metrics for the pan-cancer classification task.

Traditional ML methods

Naive Bayes [6]			Linear Regression [2]			SVM [9]			Random Forest [1]			XGBoost [3]		
Accuracy	F1-macro	F1-weighted	Accuracy	F1-macro	F1-weighted	Accuracy	F1-macro	F1-weighted	Accuracy	F1-macro	F1-weighted	Accuracy	F1-macro	F1-weighted
70.05 (1.19)	74.80 (0.94)	80.62 (1.35)	85.87 (0.22)	80.91 (0.68)	85.75 (0.23)	82.10 (0.81)	81.22 (0.87)	90.45 (0.95)	82.56 (0.73)	84.75 (1.16)	81.73 (0.88)	84.28 (0.50)	78.95 (0.63)	84.08 (0.50)

Deep Neural Network

MOGONET (2021) [10]			MMDynamic (2022) [5]			EMOGI (2021) [8]			subtype-DCC (2023) [11]			TMC (2022) [4]		
Accuracy	F1-macro	F1-weighted	Accuracy	F1-macro	F1-weighted	Accuracy	F1-macro	F1-weighted	Accuracy	F1-macro	F1-weighted	Accuracy	F1-macro	F1-weighted
86.72 (0.68)	83.47 (1.02)	86.25 (0.77)	89.83 (0.37)	87.98 (0.72)	89.80 (0.53)	85.09 (1.12)	84.72 (0.88)	87.66 (0.71)	83.58 (1.02)	81.34 (0.76)	83.29 (0.60)	89.29 (0.32)	88.42 (0.46)	89.01 (0.35)

HTML (Ours)

Accuracy	F1-macro	F1-weighted
**93.34** (0.39)	**83.45** (1.28)	**92.22** (0.49)

Table 4 Ablation study on the different modules in HTML model. We conduct an ablation study on the various modules of HTML model in the COADREAD dataset, and discover that each module plays a crucial role in the functionality of HTML.

	Full model	w/o Feature Dynamic	w/o Meth-Guided Attention	w/o Triple Contrastive Loss	w/o Modality Dynamic	w/o Dempster-Shafer Integration
Accuracy	**86.43 (6.30)**	80.41 (4.92)	77.07 (6.93)	84.21 (5.89)	80.11 (4.35)	82.50 (4.64)
F1-macro	**76.87 (10.33)**	64.41 (8.97)	46.75 (8.44)	73.88 (9.72)	57.65 (12.92)	67.11 (12.96)
F1-weighted	**84.79 (7.97)**	77.00 (6.88)	67.67 (10.10)	82.20 (7.39)	74.93 (5.86)	79.23 (7.22)
AUROC	**89.20 (5.19)**	78.25 (6.79)	83.15 (5.39)	87.64 (5.73)	69.91 (6.06)	79.46 (6.77)
AUPRC	**86.00 (9.92)**	72.20 (5.08)	79.08 (6.14)	85.17 (8.05)	67.80 (5.89)	77.45 (5.69)

Table 5 Detailed hyperparameter selection of HTML model. We conduct a grid search to determine the optimal hyperparameters for HTML in each dataset, and subsequently report the best hyperparameters.

Dataset	hidden_dim	train_epoch	dropout_rate	weight_decay	l1_lambda	learning rate
COADREAD	1,000	2000	0.5	1.00E-03	1.00E-04	1.00E-04
ESCA	500	500	0.5	1.00E-03	1.00E-04	1.00E-04
GBMLGG	1000	2000	0.5	1.00E-03	1.00E-04	5.00E-05
SARC	1000	2000	0.5	1.00E-03	1.00E-04	1.00E-04
STAD	500	500	0.5	1.00E-03	1.00E-04	1.00E-04
STES	1000	2000	0.5	1.00E-03	1.00E-04	1.00E-04
THCA	1000	2000	0.5	1.00E-03	1.00E-04	1.00E-04
UCEC	1000	2000	0.5	1.00E-03	1.00E-04	2.00E-04
BRCA	1000	2000	0.5	1.00E-03	1.00E-04	1.00E-04
LGG	1000	2000	0.5	1.00E-03	1.00E-04	2.00E-04
KIPAN	1000	2000	0.5	1.00E-03	1.00E-04	1.00E-04
ROSMAP	1000	2000	0.5	1.00E-03	1.00E-04	1.00E-04

Table 6 Result comparison under different omics combination settings. We test all possible combinations of multi-omics inputs in the COADREAD dataset and examin their corresponding classification performance.

	meth	mRNA	miRNA	meth+mRNA	meth+miRNA	mRNA+miRNA	meth+mRNA +miRNA
Accuracy	82.09(5.02)	85.84(4.07)	74.19(5.82)	87.67(3.34)	86.28(3.56)	86.05(4.35)	**87.91(3.57)**
F1-macro	50.80(4.70)	56.70(3.59)	48.79(5.50)	57.40(2.73)	55.77(2.98)	55.64(4.16)	**57.77(3.18)**
F1-weighted	78.94(5.66)	84.56(4.38)	71.25(6.56)	85.37(3.61)	83.80(3.83)	83.41(4.74)	**85.61(3.81)**
AUROC	80.89(3.65)	85.29(5.16)	63.55(6.27)	85.26(1.85)	84.45(1.84)	86.30(3.06)	**89.88(2.50)**
AUPRC	60.62(5.61)	**64.08(4.08)**	54.05(3.77)	63.41(3.86)	63.75(1.44)	63.71(3.14)	63.34(3.92)

Table 7 Abbreviation explanation. Abbreviations are used in our manuscript, figures and tables, this table offers an explanation of these abbreviations.

Abbreviation	Full name
COADREAD	COlorectal ADenocarcinoma and REctal ADenocarcinoma
ESCA	ESophageal CArcinoma
GBMLGG	GlioBlastoMa and Low-Grade Glioma
SARC	SARComa
STAD	STomach ADenocarcinoma
STES	STomach and ESophageal carcinoma
THCA	THyroid CArcinoma
UCEC	Uterine Corpus Endometrial Carcinoma
BRCA	BReast CArcinoma
LGG	Low-Grade Glioma
KIPAN	TCGA PAN-KIdney cohort
ROSMAP	Religious Orders Study and Memory and Aging Project
PRAD	PRostate ADenocarcinoma
HNSC	Head and Neck Squamous Cell carcinoma
LUAD	LUng ADenocarcinoma
SKCM	SKin Cutaneous Melanoma
BLCA	BLadder urothelial CArcinoma
LIHC	LIver Hepatocellular Carcinoma
LUSC	LUng Squamous cell Carcinoma
KIRC	KIdney Renal clear cell Carcinoma
CESC	CErvical Squamous cell Carcinoma and endocervical adenocarcinoma
KIRP	KIdney Renal Papillary cell carcinoma
PCPG	PheochromoCytoma and ParaganGlioma
PAAD	PAncreatic ADenocarcinoma
TGCT	Testicular Germ Cell Tumors
LAML	Acute Myeloid Leukemia
THYM	THYMoma
MESO	MESOthelioma
UVM	UVeal Melanoma
ACC	AdrenoCortical Carcinoma
KICH	KIdney CHromophobe
GBM	GlioBlastoma Multiforme
UCS	Uterine CarcinoSarcoma
DLBC	Lymphoid Neoplasm Diffuse Large B-cell Lymphoma
CHOL	CHOLangiocarcinoma
OV	OVarian serous cystadenocarcinoma
COAD	COlon ADenocarcinoma
READ	REctal ADenocarcinoma
ESCC	Esophagus Squamous Cell Carcinoma
EAC	Esophagus AdenoCarcinoma nos
AST	ASTrocytoma
ODG	OligoDendroGlioma
OAC	OligoAstroCytoma
LMS	LeioMyoSarcoma
DDLPS	DeDifferentiated LiPoSarcoma
ADC	ADenoCarcinoma
IAC	Intestinal AdenoCarcinoma
EEA	Endometrioid Endometrial Adenocarcinoma
SEA	Serous Endometrial Adenocarcinoma
MSEAC	Mixed Serous and Endometrioid AdenoCarcinoma

Table 8 Biomarkers with high feature dynamic weights in the COADREAD dataset. We discover the genes that rank in the top 50 for feature dynamic weight in each disease subtype in the COADREAD dataset and highlight those genes with differences as biomarkers.

COAD			READ		
**meth**	**mRNA**	**miRNA**	**meth**	**mRNA**	**miRNA**
ELAVL2	FOXA1	hsa-mir-30e	ELAVL2	FOXA1	hsa-mir-30e
RPRM	C14orf159	hsa-mir-497	ZNF702P	C14orf159	hsa-let-7a-3
CPNE8	SDHA	hsa-mir-663b	RPRM	PRAC	hsa-mir-665
COL19A1	ATP8A1	hsa-mir-665	CPNE8	SDHA	hsa-mir-497
MGC42105	ATP5A1	hsa-let-7a-3	CPAMD8	CADPS	hsa-mir-1258
VENTX	LLGL2	hsa-mir-362	MYOM3	ATP8A1	hsa-mir-127
CPAMD8	MYO5C	hsa-let-7i	MIR1908	MYO5C	hsa-mir-362
ZNF702P	STAT3	hsa-mir-1296	VENTX	GAK	hsa-mir-551b
LPHN2	ANKRD22	hsa-mir-624	PPBPL2	ANKRD22	hsa-mir-30a
MYOM3	CADPS	hsa-mir-96	SHISA3	LLGL2	hsa-mir-663b
SHISA3	ALDH2	hsa-mir-1308	SNORD5	SYNC	hsa-mir-652
LOC80154	FLII	hsa-mir-107	LOC80154	PGAM1	hsa-mir-143
SFRP4	ELAC2	hsa-mir-382	COL19A1	FAM129B	hsa-mir-624
PRAMEF21	GAK	hsa-mir-411	DDIT4L	DDX6	hsa-mir-937
HBZ	FSCN1	hsa-mir-143	LPHN2	ATP5A1	hsa-mir-96
SNORD5	LRBA	hsa-mir-532	LOC399753	IRF2BP1	hsa-mir-1296
AMH	IRF2BP1	hsa-mir-127	OCM2	FLII	hsa-mir-532
FADS1	ATP8B1	hsa-mir-937	MGC42105	ALDH2	hsa-mir-503
LOC399753	VOPP1	hsa-mir-30a	EREG	LRBA	hsa-mir-376a-2
PCK1	FAM38A	hsa-mir-551b	SFRP4	PIM3	hsa-mir-382
LRMP	HSD3B7	hsa-mir-652	SIX1	TMEM8B	hsa-mir-129-1
MIR1908	PRAC	hsa-mir-184	HOXC6	ATP8B1	hsa-mir-184
DTX3	FAM129B	hsa-mir-1258	AMH	HSD3B7	hsa-mir-658
CEACAM6	PIM3	hsa-mir-376a-2	FADS1	ELAC2	hsa-mir-196a-2
PPBPL2	TP53I11	hsa-mir-24-1	PCK1	FSCN1	hsa-mir-1308
TCF4	DOT1L	hsa-mir-500	ZNF461	VOPP1	hsa-let-7i
SIX1	PGAM1	hsa-mir-653	DTX3	TAPBP	hsa-mir-765
GOLT1A	MAP1S	hsa-mir-658	FSTL5	NRARP	hsa-mir-1201
HOXC6	TAPBP	hsa-mir-129-1	PI15	STAT3	hsa-mir-107
HIST1H2BH	TNKS1BP1	hsa-mir-597	CLVS1	MYO18A	hsa-mir-653
CDH7	MYO18A	hsa-let-7f-2	NKX2-8	DOT1L	hsa-mir-24-1
CLVS1	BCR	hsa-mir-153-2	PRAMEF21	PKM2	hsa-mir-500
EREG	SYNC	hsa-mir-101-2	CEACAM6	GALK2	hsa-let-7f-2
GC	PKM2	hsa-mir-503	WIPF3	BMP4	hsa-mir-548l
GLT8D2	NRARP	hsa-let-7b	IL20RA	CCDC85C	hsa-mir-541
VAMP5	DDX6	hsa-mir-1185-1	SDS	MAP1S	hsa-mir-1295
OCM2	SLC38A10	hsa-mir-196a-2	VAMP5	ZFP36L2	hsa-mir-125a
OXTR	BTBD6	hsa-mir-548l	CDH7	FAM38A	hsa-mir-514-1
FLJ39609	GALK2	hsa-mir-320d-1	GC	SLC25A39	hsa-mir-181c
UGT2B10	TMEM129	hsa-mir-24-2	HIST1H2BH	ACTG1	hsa-mir-153-2
NKX2-8	STRN4	hsa-mir-135a-1	GOLT1A	BCR	hsa-mir-597
WIPF3	ENO1	hsa-mir-1201	NRAP	SLC38A10	hsa-mir-550-1
IL32	TMEM8B	hsa-mir-550-1	GLT8D2	TP53I11	hsa-mir-187
PI15	SGSM3	hsa-mir-134	GPR150	SGSM3	hsa-mir-320d-1
C10orf67	DGKQ	hsa-mir-1277	CFHR1	ENO1	hsa-let-7b
IL12RB2	CCDC85C	hsa-mir-585	TRIM77	SLC37A1	hsa-mir-194-1
FLT3	ACTG1	hsa-mir-153-1	IL32	PLAUR	hsa-mir-153-1
IL20RA	CPE	hsa-mir-17	C10orf67	CPE	hsa-mir-191
SDS	SLC9A2	hsa-mir-125a	HBZ	SPON1	hsa-mir-629
SLC22A24	BMP4	hsa-mir-1248	STC1	SLC9A2	hsa-mir-24-2

Table 9 Biomarkers with high feature dynamic weights in the ESAC dataset. We discover the genes that rank in the top 50 for feature dynamic weight in each disease subtype in the ESCA dataset and highlight those genes with differences as biomarkers.

‘ADC			IAC		
**meth**	**mRNA**	**miRNA**	**meth**	**mRNA**	**miRNA**
FXYD7	OLFM4	hsa-mir-377	TNFSF13B	OLFM4	hsa-mir-509-2
TNFSF13B	SLC29A2	hsa-mir-615	FXYD7	SLC29A2	hsa-mir-377
RNF135	BTG1	hsa-mir-509-2	GAS2L3	FN3K	hsa-mir-615
GAS2L3	ERGIC3	hsa-mir-184	FRG1B	BTG1	hsa-mir-580
HIST1H2BG	GOLGA7	hsa-mir-580	PTPN13	GOLGA7	hsa-mir-184
HES7	FN3K	hsa-mir-196a-2	HIST1H2BG	KCTD3	hsa-mir-1251
ZNF649	CNTNAP2	hsa-mir-1277	ZNF649	POM121	hsa-mir-7-3
ZNF714	DCTD	hsa-mir-607	KCNC3	GLB1L2	hsa-mir-196a-2
KBTBD7	PDZD11	hsa-mir-103-1	MSI1	HLA-DRA	hsa-mir-1274b
PTPN13	KCTD3	hsa-mir-1274b	KBTBD7	RALGAPA2	hsa-mir-7-1
BBS10	MED28	hsa-mir-1251	TCN2	CNTNAP2	hsa-mir-23b
NBPF3	HLA-DRA	hsa-mir-454	ADAL	KPNA2	hsa-mir-887
FRG1B	GLB1L2	hsa-mir-197	CXCL2	ERGIC3	hsa-mir-1277
LOC100286793	PRPF19	hsa-mir-7-3	BBS10	MED28	hsa-mir-607
FLJ39739	RALGAPA2	hsa-mir-574	ZNF83	CARS2	hsa-mir-339
CXCL2	CARS2	hsa-mir-642a	CDC42EP3	DCTD	hsa-mir-100
LCMT2	POM121	hsa-mir-1307	HES7	CANT1	hsa-mir-3913-1
OGFRL1	HLA-DOA	hsa-mir-636	LOC100286793	BPTF	hsa-mir-548f-1
ADAL	NOTCH2	hsa-mir-23b	NBPF3	ZNF703	hsa-mir-3909
LOC100190940	RAB40B	hsa-mir-376b	RNF135	PDZD11	hsa-mir-3193
TCN2	DDX6	hsa-mir-190b	OGFRL1	RAB40B	hsa-mir-197
CDC42EP3	GM2A	hsa-mir-106b	TMEM106A	NOTCH2	hsa-mir-1307
ZDBF2	ZNF770	hsa-mir-887	SULT1B1	ITGA6	hsa-mir-766
SCN3B	EIF1AX	hsa-mir-25	ZNF714	HLA-DPB1	hsa-mir-25
KCNC3	ZNF703	hsa-mir-339	ACE	UNC13B	hsa-mir-636
HOXB7	CANT1	hsa-mir-100	FLJ39739	MLPH	hsa-mir-574
ACE	CDCA4	hsa-mir-18a	SCN3B	PRPF19	hsa-mir-489
C17orf51	KPNA2	hsa-mir-627	ZDBF2	PTPRF	hsa-mir-190b
MSI1	UNC13B	hsa-mir-3193	CYP39A1	ZNF770	hsa-mir-103-1
RPL39L	HLA-DPB1	hsa-mir-328	RND2	COPA	hsa-mir-1288
ZNF83	HDAC1	hsa-let-7f-2	CCDC11	HLA-DOA	hsa-mir-18a
CCDC11	CIITA	hsa-mir-766	HOXB7	DDX6	hsa-mir-642a
ACBD7	COPA	hsa-mir-374b	C17orf51	CIITA	hsa-mir-145
HIST1H3J	MLPH	hsa-mir-548f-1	PSTPIP2	DCK	hsa-mir-106b
KIT	C1orf144	hsa-mir-3909	SLC37A2	GM2A	hsa-mir-376b
LAPTM4B	RHOA	hsa-mir-545	NCRNA00152	RACGAP1	hsa-mir-551a
LOC80154	PIGO	hsa-mir-28	MDS2	PIGO	hsa-mir-28
SLC37A2	SPATA20	hsa-mir-33b	LCMT2	EIF1AX	hsa-mir-454
EVX1	ANKS6	hsa-mir-1288	ACBD7	SPATA20	hsa-mir-378
NSUN7	TOMM20	hsa-mir-3913-1	MYCNOS	CTBP2	hsa-mir-545
SLFN11	PTPRF	hsa-mir-149	CCDC15	YIPF3	hsa-mir-627
TMEM106A	ARPP19	hsa-mir-3130-1	SLFN12	RRM2B	hsa-mir-3605
BATF3	DCK	hsa-mir-1537	LAPTM4B	C9orf152	hsa-mir-15b
PSTPIP2	IER5L	hsa-mir-3605	EVX1	C1orf43	hsa-mir-3140
SLFN12	UBE2I	hsa-mir-7-1	B3GALT4	CDCA4	hsa-mir-33b
MYCNOS	MTA2	hsa-mir-145	NTN3	TRNP1	hsa-mir-582
CYP39A1	ITGA6	hsa-mir-3140	SLFN11	ANKS6	hsa-mir-34a
SULT1B1	TRNP1	hsa-mir-1224	LY75	HDAC1	hsa-mir-98
RND2	BPTF	hsa-mir-497	SLC1A4	INADL	hsa-mir-182
ZNF844	CDK2AP1	hsa-mir-15b	LOC100190940	UBE2I	hsa-mir-497

Table 10 Biomarkers with high feature dynamic weights in the GBMLGG dataset. We discover the genes that rank in the top 50 for feature dynamic weight in each disease subtype in the GBMLGG dataset and highlight those genes with differences as biomarkers.

AST			ODG			OAC		
**meth**	**mRNA**	**miRNA**	**meth**	**mRNA**	**miRNA**	**meth**	**mRNA**	**miRNA**
ZNF334	SLAIN1	hsa-mir-376a-1	MIR320C1	KCNJ11	hsa-mir-548j	ZNF334	FAM123C	hsa-mir-365-1
MIR210	DKFZp686O24166	hsa-mir-548j	ZNF334	FAM123C	hsa-mir-376a-1	HIST1H2AG	CRTAC1	hsa-mir-548j
HIST1H2AG	SEZ6L2	hsa-mir-320a	HIST1H2AG	DKFZp686O24166	hsa-mir-135a-1	MIR320C1	KCNJ11	hsa-mir-376a-1
PCDH20	CRTAC1	hsa-mir-365-1	BLVRB	FAM155A	hsa-mir-365-1	TUSC1	SRXN1	hsa-mir-3690
MIR320C1	FAM123A	hsa-mir-3690	MIR210	CRTAC1	hsa-mir-885	FLJ11235	FAM155A	hsa-mir-320a
LOC388242	PTPRN2	hsa-mir-200c	C2orf67	SLAIN1	hsa-mir-3690	HIST1H2AH	ATRX	hsa-mir-135a-1
HIST1H2AH	KCNJ11	hsa-mir-3124	REST	ATOH8	hsa-mir-320a	BLVRB	PTPRN2	hsa-mir-1295
MIR1469	FAM155A	hsa-mir-181a-1	REC8	ABTB2	hsa-mir-200c	LOC388242	DKFZp686O24166	hsa-mir-3124
FLJ11235	SRXN1	hsa-mir-135a-1	ATL3	PCDHA1	hsa-mir-590	C9orf40	FAM123A	hsa-mir-539
C2orf67	HR	hsa-mir-199a-1	PCYOX1	SCARB1	hsa-mir-223	FLJ45983	KIAA1409	hsa-mir-181a-1
C9orf40	BMP7	hsa-mir-639	LOC388242	MARCH4	hsa-mir-433	PCDH20	CLVS1	hsa-let-7a-3
ATL3	CLVS1	hsa-mir-193a	FOXD1	SRXN1	hsa-mir-346	REST	BMP7	hsa-mir-193a
REST	RGAG4	hsa-mir-889	PCDH20	HR	hsa-mir-1295	PRR24	ABTB2	hsa-mir-211
PLOD2	LOC283174	hsa-mir-543	MAN2A1	FAM123A	hsa-mir-181a-1	MIR210	MARCH4	hsa-mir-1277
BLVRB	KIAA1409	hsa-mir-3177	FLJ11235	KIAA1409	hsa-mir-199a-1	REC8	ATOH8	hsa-mir-885
PCYOX1	FAM123C	hsa-mir-514-1	SYCE2	BMP7	hsa-mir-639	SFT2D2	LOC150622	hsa-mir-3177
LIPT2	ABTB2	hsa-mir-346	HIST1H2AH	SEZ6L2	hsa-mir-301a	PLOD2	HR	hsa-mir-639
OR4C13	MARCH4	hsa-mir-211	C9orf40	MAGEH1	hsa-mir-3124	ATL3	SLAIN1	hsa-mir-199a-1
MAFB	TUB	hsa-mir-590	MIR1469	BRPF3	hsa-mir-539	FLJ16779	SEZ6L2	hsa-mir-590
PNMAL1	ATOH8	hsa-mir-223	FLJ45983	TUB	hsa-mir-3177	C2orf67	SCG5	hsa-mir-223
FLJ45983	HIP1R	hsa-mir-885	OR4C13	C8orf46	hsa-mir-514-1	MIR1469	GCNT2	hsa-mir-153-1
INPPL1	C8orf46	hsa-mir-1277	PRR24	LOC283174	hsa-mir-193a	MLH3	RGAG4	hsa-mir-200c
HIST1H2BJ	LOC150622	hsa-mir-92a-1	FLJ32065	ATRX	hsa-mir-3909	FXYD5	SCARB1	hsa-mir-543
SYCE2	MAGEH1	hsa-mir-301a	STL	LOC150622	hsa-mir-1277	FOXD1	TMEM41B	hsa-mir-181d
LEKR1	ATRX	hsa-mir-380	HDHD3	FAM134A	hsa-mir-3187	PRRG4	FAM134A	hsa-mir-769
FOXD1	SART3	hsa-mir-769	MAFB	ABCG1	hsa-mir-153-1	STL	SART3	hsa-mir-433
ADAMTS20	SCARB1	hsa-mir-433	LOC613038	SMPD3	hsa-mir-580	SYCE2	TUB	hsa-mir-301a
STL	ATCAY	hsa-mir-185	TUSC1	SART3	hsa-mir-383	FRAT2	ATXN7L3B	hsa-mir-548s
HBQ1	GABRB3	hsa-mir-1269	PNMAL1	HIP1R	hsa-mir-1254	LOC613038	C8orf46	hsa-mir-889
FLJ16779	PCDHA1	hsa-mir-580	HIST1H2BJ	CLVS1	hsa-mir-889	HIST1H2BJ	HSP90AB1	hsa-mir-370
PRR24	VAMP4	hsa-mir-3941	ADAMTS20	HSP90AB1	hsa-mir-370	CYP51A1	BRPF3	hsa-mir-580
FOXQ1	SCG5	hsa-mir-1270-2	PLOD2	RAI1	hsa-mir-543	OR4C13	GIT1	hsa-let-7e
LOC613038	SLC7A14	hsa-mir-210	HBQ1	KCNK10	hsa-let-7e	PNMAL1	HIP1R	hsa-mir-210
SFT2D2	FAM134A	hsa-mir-1254	FRAT2	MPP2	hsa-mir-624	LIPT2	RAI1	hsa-mir-942
FXYD5	RAI1	hsa-mir-3187	LIPT2	PCDH1	hsa-mir-200b	MAFB	VAMP4	hsa-mir-200b
FLJ32065	GCNT2	hsa-mir-548o	CH25H	ATCAY	hsa-mir-211	FGF19	PCDHA1	hsa-mir-185
REC8	MPP2	hsa-let-7a-3	FGF19	SLC24A3	hsa-mir-146a	ADAMTS20	ABCG1	hsa-mir-346
PRRG4	HSP90AB1	hsa-mir-539	PRRG4	HMP19	hsa-mir-548s	LEKR1	PTCD3	hsa-mir-338
NETO2	KCNK10	hsa-mir-624	FLJ16779	SLC7A14	hsa-mir-210	PCYOX1	SMPD3	hsa-mir-1269
ABLIM3	LPPR1	hsa-let-7e	PTENP1	VAMP4	hsa-mir-769	HDHD3	GABRB3	hsa-mir-514-1
FRAT2	BAP1	hsa-mir-135a-2	LEKR1	TMEM41B	hsa-let-7a-3	GPR19	ATCAY	hsa-mir-3941
MAN2A1	ANAPC5	hsa-mir-181d	GCA	ALDH1L2	hsa-mir-1269	SEPX1	C15orf59	hsa-mir-1976
TUSC1	ABCG1	hsa-mir-370	FOXQ1	GNAI1	hsa-mir-3613	HBQ1	MPP2	hsa-mir-624
PHLDA2	GIT1	hsa-mir-3199-1	SLMO1	SF1	hsa-mir-3662	FOXQ1	ECE2	hsa-mir-3613
SEPX1	C15orf59	hsa-mir-1976	SFT2D2	RNASEH2C	hsa-mir-3200	HSPA1A	LETMD1	hsa-mir-1270-2
GCA	TMEM41B	hsa-mir-942	HIST1H1D	PTPRN2	hsa-mir-24-2	WDR69	LOC283174	hsa-mir-92a-1
ARHGEF5L	ZNF33B	hsa-mir-204	FXYD5	ATXN7L3B	hsa-mir-92a-1	CH25H	MAGEH1	hsa-mir-24-2
HIST1H1D	BRPF3	hsa-mir-146a	ABLIM3	NF1	hsa-mir-128-2	INPPL1	ALDH1L2	hsa-mir-22
IMPACT	VAPA	hsa-mir-1301	SEPX1	GCNT2	hsa-mir-2116	NETO2	FXR2	hsa-mir-135a-2
LOC113230	PCDH1	hsa-mir-128-2	WDR69	PJA1	hsa-mir-1976	HIST1H1D	PRPF6	hsa-mir-1254

Table 11 Biomarkers with high feature dynamic weights in the SARC dataset. We discover the genes that rank in the top 50 for feature dynamic weight in each disease subtype in the SARC dataset and highlight those genes with differences as biomarkers.

LMS			DDLPS			others		
**meth**	**mRNA**	**miRNA**	**meth**	**mRNA**	**miRNA**	**meth**	**mRNA**	**miRNA**
ISCA1	NME2P1	hsa-mir-19b-1	FBXO47	C11orf59	hsa-mir-139	TMEFF1	ULK1	hsa-mir-139
CASS4	NDUFS6	hsa-mir-139	TMEFF1	C19orf28	hsa-mir-206	FBXO47	C1orf43	hsa-mir-19b-1
HIST1H4E	C11orf59	hsa-mir-93	C5orf39	C1orf43	hsa-mir-152	RBM24	C22orf9	hsa-mir-365-2
TMEFF1	SEC61G	hsa-mir-3682	CASS4	NDUFS6	hsa-mir-380	CASS4	GNG5	hsa-mir-582
FBXO47	MAGED1	hsa-mir-3940	LOC399753	TNIP1	hsa-mir-1307	KLHL9	RHOC	hsa-mir-93
SNORD76	EEF1A1	hsa-mir-380	SNORA78	SLC43A3	hsa-mir-3909	MIR148A	SEC61G	hsa-mir-579
ZFP28	C1orf43	hsa-mir-579	FABP5L3	CHRDL1	hsa-mir-365-2	GBX2	NME2P1	hsa-mir-1307
TNFAIP6	LSM4	hsa-mir-548d-2	ZNF620	EEF1A1	hsa-let-7b	FABP5L3	PRELID1	hsa-mir-873
RBM24	YKT6	hsa-mir-106a	HIST1H4J	PRELID1	hsa-mir-639	ISCA1	C11orf59	hsa-mir-106a
C15orf26	PLP2	hsa-mir-206	MIR148A	ULK1	hsa-mir-19b-1	SNORD77	POMGNT1	hsa-mir-380
ZNF620	OXA1L	hsa-mir-1307	TNFAIP6	MAGED1	hsa-mir-655	MAFA	NDUFS6	hsa-mir-548d-2
FBLL1	GRN	hsa-mir-582	ISCA1	NME2P1	hsa-mir-487b	HIST1H4J	CKS1B	hsa-mir-668
MAFA	GNG5	hsa-mir-618	ZFP28	MCTS1	hsa-mir-618	IL10RB	EEF1A1	hsa-mir-584
SIX6	RDBP	hsa-mir-3909	KLHL9	YKT6	hsa-mir-877	C5orf39	MIF	hsa-mir-3682
ZNF860	ULK1	hsa-mir-651	SNORD77	EFHD2	hsa-mir-3923	ZFP28	TNIP1	hsa-let-7b
LOC399753	PRAF2	hsa-mir-655	MIR663	GNG5	hsa-mir-582	ZNF382	SLC43A3	hsa-mir-1277
FABP5L3	PFDN5	hsa-mir-639	NCRNA00116	VARS	hsa-mir-605	AKAP7	YKT6	hsa-mir-639
HAAO	KPNB1	hsa-mir-3923	TMEM106A	TMEM109	hsa-mir-3917	NCRNA00116	WHSC1	hsa-mir-205
SLC16A5	SF3A2	hsa-mir-1277	AKAP7	C22orf9	hsa-mir-1277	TNFAIP6	MAGED1	hsa-mir-206
SNORA78	NDUFB5	hsa-mir-101-1	KLF4	OXA1L	hsa-mir-195	GPR135	VARS	hsa-mir-3923
LOC646999	VARS	hsa-mir-129-1	LOC646999	NPLOC4	hsa-mir-3682	LOC399753	ASCC2	hsa-mir-152
KLHL9	CACYBP	hsa-mir-877	HIST1H1D	MED16	hsa-mir-651	SNORA78	LSM4	hsa-mir-605
C5orf39	C22orf9	hsa-mir-342	MIR193A	POMGNT1	hsa-mir-101-1	C15orf26	C1orf151	hsa-mir-129-1
MTMR11	LSM7	hsa-mir-873	GPR19	LILRB4	hsa-mir-423	MIR663	PRPF19	hsa-mir-655
GBX2	TMEM109	hsa-mir-365-2	TRIM59	MIF	hsa-mir-106a	SNORD76	GRN	hsa-mir-589
LOC80054	U2AF1	hsa-mir-1180	EGLN3	RNF167	hsa-mir-579	TRIM59	HMGN2	hsa-mir-3917
MIR148A	RHOC	hsa-mir-584	GBX2	RHOC	hsa-mir-584	TMEM106A	SF3A2	hsa-mir-618
LOC151534	DLGAP4	hsa-mir-487b	HIST1H4K	CKS1B	hsa-mir-3940	FBLL1	CACYBP	hsa-mir-146b
ZNF781	EFHD2	hsa-mir-1228	GPR135	FAM125A	hsa-mir-548d-2	SIX6	LILRB4	hsa-mir-363
TNFSF4	ADRBK1	hsa-mir-423	TRIM58	PRAF2	hsa-mir-215	HIST1H1D	OXA1L	hsa-mir-423
KLHDC9	SH3KBP1	hsa-mir-605	SNHG9	GYPC	hsa-mir-502	HOXA2	THOC5	hsa-mir-101-1
NPB	LILRB4	hsa-mir-151	LOC151534	AGTRAP	hsa-mir-1292	SNHG9	NDUFB5	hsa-mir-877
COL12A1	TSC22D3	hsa-mir-28	MAFA	KPNB1	hsa-mir-205	FAM115A	C19orf28	hsa-mir-3909
GPR135	AGTRAP	hsa-mir-205	HIST1H4E	SCNM1	hsa-mir-548k	HIST1H4K	PFDN5	hsa-mir-1976
HIST1H4J	C19orf28	hsa-mir-146b	RBM24	SEC61G	hsa-mir-1180	CYR61	SCNM1	hsa-mir-487b
ZNF382	KLHDC3	hsa-let-7b	ZNF382	RDBP	hsa-mir-342	HAAO	EFHD2	hsa-mir-342
SNX22	EXOSC4	hsa-mir-196a-1	ZNF860	ASCC2	hsa-mir-409	LOC646999	AGTRAP	hsa-mir-720
AKAP7	CCS	hsa-mir-502	HAAO	FXR1	hsa-mir-548j	KLHDC9	PRR13	hsa-mir-590
HIST1H1D	ZIC2	hsa-mir-744	CENPV	PSMB3	hsa-mir-541	HCP5	RDBP	hsa-mir-133a-2
HIST1H4K	CSK	hsa-mir-551b	HOXA2	RBM42	hsa-mir-92a-1	SLC16A5	CHRDL1	hsa-mir-28
IL10RB	GBP2	hsa-mir-1976	LOC80054	ARMC5	hsa-mir-151	FLJ13224	PLEC	hsa-mir-652
KLF4	SLC43A3	hsa-mir-652	SNORD17	FLNB	hsa-mir-518c	EGLN3	ARFGAP1	hsa-mir-651
SLCO2A1	MIF	hsa-mir-664	LOC391322	CACYBP	hsa-mir-381	SEPT10	E2F1	hsa-mir-3619
TMEM2	RPLP2	hsa-mir-548b	TBX21	CCDC72	hsa-mir-3619	MTAP	SENP3	hsa-mir-1180
CHAD	RPL38	hsa-mir-520a	TMEM2	RPN1	hsa-mir-873	MIR193A	RPN1	hsa-mir-3940
KDR	ARFGAP1	hsa-mir-3127	FBLL1	SLC2A4RG	hsa-mir-3193	ZNF781	PRAF2	hsa-mir-502
C2orf62	RBM42	hsa-mir-3934	IL10RB	SENP3	hsa-mir-518b	TBX21	SH3KBP1	hsa-mir-30a
TBX21	TNIP1	hsa-mir-940	HCP5	COX6A1	hsa-mir-589	CREG2	GYPC	hsa-mir-940
LVRN	NONO	hsa-mir-23a	ZNF781	RPS5	hsa-mir-129-1	SNORD75	HSD3B7	hsa-mir-3127
CENPV	E2F1	hsa-mir-3917	SIX6	RPL35A	hsa-mir-3934	MTMR11	PPM1G	hsa-mir-541

Table 12 Biomarkers with high feature dynamic weights in the STAD dataset. We discover the genes that rank in the top 50 for feature dynamic weight in each disease subtype in the STAD dataset and highlight those genes with differences as biomarkers.

'ADC			IAC		
**meth**	**mRNA**	**miRNA**	**meth**	**mRNA**	**miRNA**
FXYD7	OLFM4	hsa-mir-377	TNFSF13B	OLFM4	hsa-mir-509-2
TNFSF13B	SLC29A2	hsa-mir-615	FXYD7	SLC29A2	hsa-mir-377
RNF135	BTG1	hsa-mir-509-2	GAS2L3	FN3K	hsa-mir-615
GAS2L3	ERGIC3	hsa-mir-184	FRG1B	BTG1	hsa-mir-580
HIST1H2BG	GOLGA7	hsa-mir-580	PTPN13	GOLGA7	hsa-mir-184
HES7	FN3K	hsa-mir-196a-2	HIST1H2BG	KCTD3	hsa-mir-1251
ZNF649	CNTNAP2	hsa-mir-1277	ZNF649	POM121	hsa-mir-7-3
ZNF714	DCTD	hsa-mir-607	KCNC3	GLB1L2	hsa-mir-196a-2
KBTBD7	PDZD11	hsa-mir-103-1	MSI1	HLA-DRA	hsa-mir-1274b
PTPN13	KCTD3	hsa-mir-1274b	KBTBD7	RALGAPA2	hsa-mir-7-1
BBS10	MED28	hsa-mir-1251	TCN2	CNTNAP2	hsa-mir-23b
NBPF3	HLA-DRA	hsa-mir-454	ADAL	KPNA2	hsa-mir-887
FRG1B	GLB1L2	hsa-mir-197	CXCL2	ERGIC3	hsa-mir-1277
LOC100286793	PRPF19	hsa-mir-7-3	BBS10	MED28	hsa-mir-607
FLJ39739	RALGAPA2	hsa-mir-574	ZNF83	CARS2	hsa-mir-339
CXCL2	CARS2	hsa-mir-642a	CDC42EP3	DCTD	hsa-mir-100
LCMT2	POM121	hsa-mir-1307	HES7	CANT1	hsa-mir-3913-1
OGFRL1	HLA-DOA	hsa-mir-636	LOC100286793	BPTF	hsa-mir-548f-1
ADAL	NOTCH2	hsa-mir-23b	NBPF3	ZNF703	hsa-mir-3909
LOC100190940	RAB40B	hsa-mir-376b	RNF135	PDZD11	hsa-mir-3193
TCN2	DDX6	hsa-mir-190b	OGFRL1	RAB40B	hsa-mir-197
CDC42EP3	GM2A	hsa-mir-106b	TMEM106A	NOTCH2	hsa-mir-1307
ZDBF2	ZNF770	hsa-mir-887	SULT1B1	ITGA6	hsa-mir-766
SCN3B	EIF1AX	hsa-mir-25	ZNF714	HLA-DPB1	hsa-mir-25
KCNC3	ZNF703	hsa-mir-339	ACE	UNC13B	hsa-mir-636
HOXB7	CANT1	hsa-mir-100	FLJ39739	MLPH	hsa-mir-574
ACE	CDCA4	hsa-mir-18a	SCN3B	PRPF19	hsa-mir-489
C17orf51	KPNA2	hsa-mir-627	ZDBF2	PTPRF	hsa-mir-190b
MSI1	UNC13B	hsa-mir-3193	CYP39A1	ZNF770	hsa-mir-103-1
RPL39L	HLA-DPB1	hsa-mir-328	RND2	COPA	hsa-mir-1288
ZNF83	HDAC1	hsa-let-7f-2	CCDC11	HLA-DOA	hsa-mir-18a
CCDC11	CIITA	hsa-mir-766	HOXB7	DDX6	hsa-mir-642a
ACBD7	COPA	hsa-mir-374b	C17orf51	CIITA	hsa-mir-145
HIST1H3J	MLPH	hsa-mir-548f-1	PSTPIP2	DCK	hsa-mir-106b
KIT	C1orf144	hsa-mir-3909	SLC37A2	GM2A	hsa-mir-376b
LAPTM4B	RHOA	hsa-mir-545	NCRNA00152	RACGAP1	hsa-mir-551a
LOC80154	PIGO	hsa-mir-28	MDS2	PIGO	hsa-mir-28
SLC37A2	SPATA20	hsa-mir-33b	LCMT2	EIF1AX	hsa-mir-454
EVX1	ANKS6	hsa-mir-1288	ACBD7	SPATA20	hsa-mir-378
NSUN7	TOMM20	hsa-mir-3913-1	MYCNOS	CTBP2	hsa-mir-545
SLFN11	PTPRF	hsa-mir-149	CCDC15	YIPF3	hsa-mir-627
TMEM106A	ARPP19	hsa-mir-3130-1	SLFN12	RRM2B	hsa-mir-3605
BATF3	DCK	hsa-mir-1537	LAPTM4B	C9orf152	hsa-mir-15b
PSTPIP2	IER5L	hsa-mir-3605	EVX1	C1orf43	hsa-mir-3140
SLFN12	UBE2I	hsa-mir-7-1	B3GALT4	CDCA4	hsa-mir-33b
MYCNOS	MTA2	hsa-mir-145	NTN3	TRNP1	hsa-mir-582
CYP39A1	ITGA6	hsa-mir-3140	SLFN11	ANKS6	hsa-mir-34a
SULT1B1	TRNP1	hsa-mir-1224	LY75	HDAC1	hsa-mir-98
RND2	BPTF	hsa-mir-497	SLC1A4	INADL	hsa-mir-182
ZNF844	CDK2AP1	hsa-mir-15b	LOC100190940	UBE2I	hsa-mir-497

Table 13 Biomarkers with high feature dynamic weights in the STES dataset. We discover the genes that rank in the top 50 for feature dynamic weight in each disease subtype in the STES dataset and highlight those genes with differences as biomarkers.

ESCC			EAC		
**meth**	**mRNA**	**miRNA**	**meth**	**mRNA**	**miRNA**
ZNF323	BCL6	hsa-mir-95	ZNF323	IFI16	hsa-mir-95
VAMP5	IFI16	hsa-let-7a-2	PRR15	FAT2	hsa-mir-1288
MIR192	RUNX3	hsa-mir-548o	HNF1B	KCTD15	hsa-mir-1307
LXN	ELOVL5	hsa-mir-204	MIR149	RAB12	hsa-let-7a-2
PRR15	BCL11B	hsa-mir-181b-2	CD2AP	CLIP4	hsa-mir-7-1
MIR149	TP63	hsa-mir-3136	GPR81	ELOVL5	hsa-mir-1538
GPR81	FSCN1	hsa-mir-1538	PPP1R15A	TP63	hsa-mir-3913-1
BCL2L1	KCTD15	hsa-mir-1304	TMEM139	VANGL2	hsa-mir-143
CD2AP	FAT2	hsa-mir-3176	SPHK2	FSCN1	hsa-mir-1266
HOXA6	SPSB1	hsa-mir-101-2	MIR148A	DGKA	hsa-mir-511-2
KRT7	TUSC3	hsa-mir-1288	BCL2L1	BCL6	hsa-mir-3136
CCDC102A	MARK4	hsa-mir-511-2	B3GNT7	RUNX3	hsa-mir-21
B3GNT7	MSN	hsa-mir-3188	CCDC102A	A4GALT	hsa-mir-636
DOK4	GLI3	hsa-mir-940	LCE2D	MDFIC	hsa-mir-23c
TMEM139	KRI1	hsa-mir-519a-2	DOK4	ABCC1	hsa-mir-412
ETNK1	TMEM189	hsa-mir-377	POSTN	TPBG	hsa-mir-3176
LOC80054	ZCCHC11	hsa-mir-7-1	HOXA6	SEMA4A	hsa-mir-3613
KRT71	CLIP4	hsa-mir-23c	BST1	ALS2CR4	hsa-mir-664
IL18	LDHB	hsa-mir-143	C8orf4	HSPB1	hsa-mir-3188
TMED6	HSPA4	hsa-mir-3913-1	GFM1	TUSC3	hsa-mir-519a-2
BCL2L15	RAB12	hsa-mir-1292	VAMP5	KIAA0922	hsa-mir-548o
FZD5	TPBG	hsa-mir-664	TMED6	ARL2BP	hsa-mir-192
ME3	ABCC1	hsa-mir-636	TMPRSS2	DFNA5	hsa-mir-760
DEFB107A	ABCF3	hsa-mir-1307	BCL2L15	BCL11B	hsa-mir-1304
MIR148A	ARL2BP	hsa-mir-3613	DDIT3	LDHB	hsa-mir-181b-2
C1orf55	CEP68	hsa-mir-30c-2	FAM153A	DRAP1	hsa-mir-663
BST1	HSPB1	hsa-mir-552	CFTR	GALNTL4	hsa-mir-940
C21orf96	GALNTL4	hsa-mir-641	CIDEC	IGSF3	hsa-mir-216a
PPP1R15A	RANGAP1	hsa-mir-21	G0S2	BCL2L13	hsa-mir-101-2
LOC84931	PLXNB3	hsa-mir-412	ME3	C14orf4	hsa-mir-3942
DDIT3	FZD6	hsa-mir-3912	IL18	MSN	hsa-mir-503
LGALS2	WDR91	hsa-mir-663	C21orf96	HSPA4	hsa-mir-3140
DOK1	PAK2	hsa-mir-152	OR8G2	SPSB1	hsa-mir-552
SOX9	BCL2L13	hsa-mir-760	TBC1D23	HK1	hsa-mir-377
SPHK2	B4GALNT1	hsa-mir-128-1	KRT7	NFE2L1	hsa-mir-204
HNF1B	VANGL2	hsa-mir-1266	SEPP1	PLSCR3	hsa-mir-641
RAB20	CNN2	hsa-mir-103-1	MIR600	FADD	hsa-mir-125b-1
KAZALD1	SEMA4A	hsa-mir-16-1	DEFB107A	IRX4	hsa-mir-1275
CIDEC	TBL1XR1	hsa-mir-320c-1	RAB20	STON2	hsa-mir-320c-1
SEL1L3	HK1	hsa-mir-140	LBX2	MCC	hsa-mir-1254
GALC	NLRP1	hsa-mir-153-2	LXN	ZCCHC11	hsa-mir-802
LOC553137	RECQL	hsa-mir-503	IFITM2	RBM19	hsa-mir-3912
TMPRSS2	PLCH2	hsa-mir-802	HOXC10	HOMER3	hsa-mir-487a
MIR215	KEAP1	hsa-mir-216a	MPST	B4GALNT1	hsa-mir-421
C8orf4	MCC	hsa-mir-653	CMTM7	MRPL51	hsa-mir-185
CASS4	HCFC1R1	hsa-mir-3138	KCTD14	FAM83C	hsa-mir-877
C11orf86	KIAA0922	hsa-mir-3140	RNF126P1	PTPN1	hsa-mir-153-2
S100P	TNKS1BP1	hsa-mir-1293	LOC553137	FZD6	hsa-let-7a-3
RCOR1	AMZ2	hsa-mir-378	KAZALD1	TNKS1BP1	hsa-mir-152
HOXC10	MDFIC	hsa-mir-9-1	SLC29A1	PKP1	hsa-mir-140

Table 14 Biomarkers with high feature dynamic weights in the THCA dataset. We discover the genes that rank in the top 50 for feature dynamic weight in each disease subtype in the THCA dataset and highlight those genes with differences as biomarkers.

Usual type			Unusual type		
**meth**	**mRNA**	**miRNA**	**meth**	**mRNA**	**miRNA**
ASIP	PDE8B	hsa-mir-508	C20orf195	PDE8B	hsa-mir-508
RNF208	GNA14	hsa-mir-324	ASIP	FLT1	hsa-mir-324
CHRM4	PBX3	hsa-mir-361	CLCF1	LOC286002	hsa-mir-361
NTS	TPMT	hsa-mir-28	SLC22A20	PBX3	hsa-mir-30c-2
SLC22A20	FLT1	hsa-mir-629	ALOX15B	TPMT	hsa-mir-629
CLCF1	WASF3	hsa-mir-30c-2	SLC27A6	WASF3	hsa-mir-19a
CAD	ST6GAL2	hsa-mir-92b	CEBPE	ARL5A	hsa-mir-28
C20orf195	LOC286002	hsa-mir-19a	AKNA	ANKRD46	hsa-mir-548o
MSI1	MOAP1	hsa-mir-551a	IER3	ZADH2	hsa-mir-3157
CRABP2	PDE7B	hsa-mir-1296	RNF208	ST6GAL2	hsa-mir-551a
LOC100128071	ZADH2	hsa-mir-877	CSF2	SNRNP25	hsa-mir-328
SLC27A6	ARL5A	hsa-mir-301b	CRABP2	GNA14	hsa-mir-1296
CSF2	SLC39A14	hsa-mir-548o	LIMK1	LYRM7	hsa-mir-511-2
IER3	FAHD2A	hsa-mir-3157	CHRM4	ATP5H	hsa-mir-301b
RICS	CFL2	hsa-mir-605	MAPK13	SLC39A14	hsa-mir-130b
EGOT	ANKRD46	hsa-mir-2114	EGOT	CFL2	hsa-mir-210
ALOX15B	STC2	hsa-mir-200a	SPATC1	MOAP1	hsa-mir-627
TSSK3	SNRNP25	hsa-mir-299	ALDOC	PDE7B	hsa-mir-129-2
DNAH1	MLEC	hsa-mir-129-2	EMP1	FAHD2A	hsa-mir-95
SEPP1	EDNRB	hsa-mir-424	LOC100128071	AAGAB	hsa-mir-424
FBXO27	IQGAP2	hsa-mir-660	MIR525	EDNRB	hsa-mir-7-2
CEBPE	NDUFA5	hsa-mir-526b	FBXO27	STC2	hsa-mir-449b
IL2RA	CMTM4	hsa-mir-3117	NTS	CMTM4	hsa-mir-514-3
EMP1	ATP5H	hsa-mir-210	MSI1	FAM117A	hsa-mir-299
MIR525	MAOA	hsa-mir-433	DUSP6	MED29	hsa-mir-605
RAG2	LYRM7	hsa-mir-511-2	TSSK3	IQGAP2	hsa-mir-92b
PXMP4	NUDT9	hsa-mir-130b	CAD	CD24	hsa-mir-186
AKNA	PRKACB	hsa-mir-328	PXMP4	PIGH	hsa-mir-138-1
GBP2	CD24	hsa-mir-217	TAGLN	NUDT9	hsa-mir-148a
SLC23A1	GPD1L	hsa-mir-16-2	CAMK2N1	NDUFA5	hsa-mir-877
GATSL1	BTNL9	hsa-mir-514-3	C5AR1	PRKACB	hsa-mir-3926-1
MAPK13	ABHD10	hsa-mir-7-2	TRIP4	BTNL9	hsa-mir-190b
C11orf74	PIGH	hsa-mir-627	HIST1H3H	GPD1L	hsa-mir-2114
CAMK2N1	MED29	hsa-mir-138-1	RAG2	MAOA	hsa-mir-660
LIMK1	DNAJB9	hsa-mir-132	C11orf74	MLEC	hsa-mir-200a
TUBB6	AAGAB	hsa-mir-9-1	RICS	DNAJB9	hsa-mir-1307
C5AR1	PCBP2	hsa-mir-346	PTGIS	WSB2	hsa-mir-217
KLHDC5	APOC1	hsa-mir-95	NCRNA00152	ABHD10	hsa-mir-1270-1
ALDOC	FAM171B	hsa-mir-541	IL2RA	CNDP2	hsa-mir-506
HIST1H3H	FAM117A	hsa-mir-3610	LTF	TMEM80	hsa-mir-135a-1
DUSP6	CCDC149	hsa-mir-103-1	SLC6A17	PCBP2	hsa-mir-10a
OR6Q1	ESAM	hsa-mir-199a-1	TGIF2	ACAD8	hsa-mir-9-1
SCGB3A1	UBE2O	hsa-mir-3605	C1orf133	ESAM	hsa-mir-103-1
MAG	ACAD8	hsa-mir-3936	SEPP1	KIAA1191	hsa-mir-301a
FBLIM1	TMEM129	hsa-mir-3200	GBP2	RPN1	hsa-mir-513c
SPATC1	PNPLA4	hsa-mir-3917	GATSL1	TMEM129	hsa-mir-3200
RGL3	CNDP2	hsa-mir-3944	KLF6	MYCT1	hsa-mir-181a-2
TGIF2	MYCT1	hsa-mir-106b	SNORD114-1	UBE2O	hsa-mir-3610
FLOT1	MPPE1	hsa-mir-139	LOC151534	TRIM58	hsa-mir-511-1
MIR21	TRIM58	hsa-mir-3676	MORN3	PNPLA4	hsa-mir-2117

Table 15 Biomarkers with high feature dynamic weights in the UCEC dataset. We discover the genes that rank in the top 50 for feature dynamic weight in each disease subtype in the UCEC dataset and highlight those genes with differences as biomarkers.

EEA			SEA			MSEAC		
**meth**	**mRNA**	**miRNA**	**meth**	**mRNA**	**miRNA**	**meth**	**mRNA**	**miRNA**
LRRC41	MX2	hsa-mir-532	UQCRH	TRO	hsa-mir-532	C5orf39	TRO	hsa-mir-1277
FAM174B	L1CAM	hsa-mir-1913	GUCY2G	MX2	hsa-mir-1277	UQCRH	MX2	hsa-mir-532
GUCY2G	TRO	hsa-mir-320a	C5orf39	L1CAM	hsa-mir-320a	FAM174B	L1CAM	hsa-mir-200b
MYCT1	DDX27	hsa-mir-139	TOMM40L	MAGEH1	hsa-mir-1913	TOMM40L	MAGEH1	hsa-mir-139
TRIM59	CLDN6	hsa-mir-377	MIR1283-2	CLDN6	hsa-mir-1271	DEPDC6	MAL2	hsa-mir-23c
TOMM40L	SIRT5	hsa-mir-199b	HPS4	DDX27	hsa-mir-139	MIR1283-2	DDX27	hsa-mir-539
LOC150381	MAGEH1	hsa-mir-556	LRRC41	BUB1	hsa-mir-377	ATF7	BUB1	hsa-mir-18a
DEPDC6	BUB1	hsa-mir-1277	SIGLECP3	SIRT5	hsa-mir-199b	LRRC41	NUDT15	hsa-mir-1271
HPS4	MAL2	hsa-mir-181d	MYCT1	MAL2	hsa-mir-1224	TMEM161B	SIRT5	hsa-mir-1910
MIR498	WDR43	hsa-mir-30e	HIST1H4D	GLDC	hsa-mir-765	MIR516A1	FOXP4	hsa-mir-3913-1
UQCRH	FOXP4	hsa-mir-128-2	DEPDC6	CDR2L	hsa-mir-137	GUCY2G	MRPL3	hsa-mir-3145
CNOT6L	CDR2L	hsa-mir-585	KRTCAP3	NUDT15	hsa-mir-200b	LOC100271722	NOL11	hsa-let-7g
ATF7	CHRAC1	hsa-mir-181c	C10orf41	MRPL3	hsa-mir-548t	HIST1H4D	CDV3	hsa-mir-126
SIGLECP3	NOL11	hsa-mir-1271	ATF7	NOL11	hsa-mir-503	SLC18A1	CDR2L	hsa-mir-181d
NPBWR2	MRPL3	hsa-mir-485	CCDC61	CHRAC1	hsa-mir-585	OR2T4	GLDC	hsa-mir-3199-1
COASY	NUDT15	hsa-mir-559	TMEM161B	FOXP4	hsa-mir-3145	SIGLECP3	PTP4A3	hsa-mir-3117
C5orf39	CDV3	hsa-mir-539	TRIM59	WDR43	hsa-mir-3117	TTC9	CHRAC1	hsa-mir-2276
TTC9	NCL	hsa-mir-200b	FAM174B	PBX2	hsa-mir-3684	OR10T2	NCL	hsa-mir-518e
CCDC61	GLDC	hsa-mir-765	UBLCP1	CDV3	hsa-mir-3174	C10orf41	CLDN6	hsa-mir-3198
MECOM	PBX2	hsa-mir-891a	SERINC5	NCL	hsa-mir-3191	KLHDC7A	PBX2	hsa-mir-377
KRT8	HNRNPUL2	hsa-mir-548t	OR2T4	NDC80	hsa-mir-539	FAM167A	HNRNPUL2	hsa-mir-765
MIR1283-2	SENP2	hsa-mir-296	NPBWR2	RPSAP9	hsa-mir-3944	KRTCAP3	RPSAP9	hsa-mir-27b
HIST1H4D	NDC80	hsa-mir-3117	BRCA1	SENP2	hsa-mir-380	ARID1A	PTBP2	hsa-mir-374b
SERINC5	LOC152217	hsa-mir-187	HNRNPF	LOC152217	hsa-mir-18a	F2RL1	LOC152217	hsa-mir-320a
TMEM161B	IGF2BP2	hsa-mir-1266	COASY	PTP4A3	hsa-let-7g	RNF222	SENP2	hsa-mir-143
OR10T2	GTF3C2	hsa-mir-2276	TTC9	TPX2	hsa-mir-128-2	PSIMCT-1	C6orf62	hsa-mir-659
KRTCAP3	RPSAP9	hsa-mir-3684	MIR498	AIF1L	hsa-mir-2276	MYCT1	TPX2	hsa-mir-503
DMC1	MFF	hsa-mir-3174	OR10T2	IGF2BP2	hsa-mir-187	ZNF175	NDC80	hsa-mir-1913
MIR7-1	PTP4A3	hsa-mir-16-1	OR4C46	GTF3C2	hsa-mir-3198	OR2W3	WDR43	hsa-mir-431
C10orf41	TPX2	hsa-mir-574	MIR375	PTBP2	hsa-mir-3667	HNRNPF	BEX4	hsa-mir-199b
MIR1826	PTBP2	hsa-mir-548o	LOC150381	HNRNPUL2	hsa-mir-1292	LOC150381	IGF2BP2	hsa-mir-296
MIR516A1	CLCF1	hsa-mir-651	LOC285370	MFF	hsa-mir-296	LOC400931	ITCH	hsa-mir-3619
PCYOX1	BEX4	hsa-mir-337	MIR516A1	C6orf62	hsa-mir-556	UGT2B15	CLCF1	hsa-mir-551b
C8orf4	C6orf62	hsa-mir-1292	KRT8	BEX4	hsa-mir-559	HM13	GTF3C2	hsa-mir-136
SLC20A1	ITCH	hsa-mir-3199-1	F2RL1	EPHB2	hsa-mir-548o	KRT8	MFF	hsa-mir-4326
MIR375	CHERP	hsa-mir-3926-1	MECOM	SKIL	hsa-mir-935	CNOT6L	AK2	hsa-mir-574
RNF222	FAM127A	hsa-mir-15b	CNOT6L	ITCH	hsa-mir-181c	CCDC61	CHERP	hsa-mir-559
PRAMEF21	AIF1L	hsa-mir-3198	DLX5	CLCF1	hsa-mir-579	NME3	NXF2	hsa-mir-30a
XBP1	BIN1	hsa-mir-137	RPS3	CHERP	hsa-mir-126	C8orf4	ARHGAP23	hsa-mir-548j
BRCA1	SKIL	hsa-mir-935	XBP1	BIN1	hsa-mir-518e	SERINC5	RPS18	hsa-mir-581
F2RL1	EPHB2	hsa-mir-581	LOC100271722	FAM127A	hsa-mir-1227	RPS3	EPCAM	hsa-mir-187
PSIMCT-1	AK2	hsa-mir-1227	OR2W3	C6orf106	hsa-mir-181d	RNF41	EPHB2	hsa-mir-598
LOC285370	MAGOH	hsa-mir-1224	MIR1826	HNRNPA1L2	hsa-mir-659	LGR5	SKIL	hsa-mir-548q
OR2T4	STON2	hsa-mir-136	ZNF175	NXF2	hsa-mir-30e	OR4C46	MGAT4A	hsa-mir-651
MIR138-1	ZNF512	hsa-mir-29b-1	UGT2B15	AK2	hsa-mir-346	KLHL35	PAX8	hsa-mir-522
HNRNPF	ARHGAP23	hsa-mir-548q	KRTAP5-6	ARHGAP23	hsa-mir-23c	TMEM134	DHX36	hsa-mir-29c
RPS3	MGAT4A	hsa-mir-3667	KLHL35	STON2	hsa-mir-374b	HIST1H2BK	BIN1	hsa-mir-337
SIRPB2	C6orf106	hsa-mir-380	TEC	MGAT4A	hsa-mir-616	MECOM	MAGOH	hsa-mir-330
TREX1	NXF2	hsa-mir-579	PCYOX1	GPN1	hsa-mir-138-1	DLX5	HDAC5	hsa-mir-612
MIR549	GPN1	hsa-mir-503	SLC20A1	MAGOH	hsa-mir-891a	SIRPB2	HNRNPA1L2	hsa-mir-1306

Table 16 Biomarkers with high feature dynamic weights in the BRCA dataset. We discover the genes that rank in the top 50 for feature dynamic weight in each disease subtype in the BRCA dataset and highlight those genes with differences as biomarkers.

Normal-like		
**DNA**	**mRNA**	**miRNA**
TPSG1	C5orf39	hsa-mir-149
MAG	RPS15A	hsa-mir-187
LY6D	CR1	hsa-mir-542
LOC100128977	MIR365-1	hsa-mir-299
ISL2	OR11H12	hsa-let-7g
C1QL4	FLJ35024	hsa-mir-3926-1
CA6	ZNF671	hsa-mir-675
KRT6C	TNFSF13B	hsa-mir-3909
LOC100130148	TTC36	hsa-mir-100
TUBA3E	MIR623	hsa-mir-744
SYT9	NLRP8	hsa-mir-1307
NXNL2	B4GALT6	hsa-mir-380
CXorf61	GALT	hsa-mir-3614
PTCHD1	OR4N5	hsa-mir-3199-2
NCRNA00092	CSTA	hsa-mir-3680
ZIC1	PCDH8	hsa-mir-766
GDF5	TAL2	hsa-mir-127
RASGEF1C	CRYAB	hsa-mir-3136
KRT16	MIR124-2	hsa-mir-877
CYP4Z2P	DOK5	hsa-mir-2355
SBSN	COQ3	hsa-mir-486
OSR1	POU4F1	hsa-mir-615
C1QL2	PAPPA2	hsa-mir-939
PTPRZ1	LOC100130331	hsa-mir-541
WDR65	EGR4	hsa-mir-335
C20orf114	PRAP1	hsa-mir-934
C20orf26	SOX2	hsa-mir-184
CYP2B7P1	PSAT1	hsa-mir-488
SLC22A16	FAM124B	hsa-mir-376a-2
TMPRSS6	EN1	hsa-mir-3150b
ROPN1B	IGFBP4	hsa-mir-497
MDGA2	C19orf59	hsa-mir-30c-1
SLC6A11	DARC	hsa-mir-944
ACE2	ID4	hsa-mir-548b
KIF4B	HSPB2	hsa-mir-153-1
SPEF1	LOC145837	hsa-mir-3676
IGFALS	NES	hsa-mir-550a-1
C8orf47	ALDH1A3	hsa-mir-136
SCN2B	GAS1	hsa-mir-489
LOC84740	LOC285692	hsa-mir-550a-2
CXCL1	TLR1	hsa-mir-296
SH3GL3	SOX9	hsa-mir-889
FABP7	ZNF80	hsa-mir-1301
KCNK15	TLX3	hsa-mir-1181
TSLP	SLC30A10	hsa-mir-320b-2
AGR3	NFE2L3	hsa-mir-129-2
PCSK4	LBR	hsa-mir-320a
SOX11	IRF1	hsa-mir-3150
COL11A2	SNORD93	hsa-mir-639
RAET1L	MIR128-1	hsa-mir-605

Basal-like		
**DNA**	**mRNA**	**miRNA**
ISL2	CR1	hsa-mir-187
LOC100128977	NLRP8	hsa-let-7g
LY6D	OR11H12	hsa-mir-149
TPSG1	TAL2	hsa-mir-299
TUBA3E	GALT	hsa-mir-3926-1
MAG	MIR365-1	hsa-mir-1307
OSR1	ZNF671	hsa-mir-744
C1QL4	RPS15A	hsa-mir-3680
ZIC1	COQ3	hsa-mir-934
KRT6C	CRYAB	hsa-mir-542
LOC100130148	LOC100130331	hsa-mir-3614
SBSN	ID4	hsa-mir-136
CYP4Z2P	POU4F1	hsa-mir-3909
PTPRZ1	C5orf39	hsa-mir-3136
CYP2B7P1	PSAT1	hsa-mir-380
RASGEF1C	OR4N5	hsa-mir-548b
ROPN1B	DOK5	hsa-mir-153-1
NXNL2	CSTA	hsa-mir-127
SLC22A16	B4GALT6	hsa-mir-541
C1QL2	FLJ35024	hsa-mir-550a-1
SYT9	TTC36	hsa-mir-30c-1
NCRNA00092	PRAP1	hsa-mir-639
CA6	IRF1	hsa-mir-675
CXorf61	EN1	hsa-mir-296
SPEF1	DARC	hsa-mir-376a-2
ACE2	SOX2	hsa-mir-103-2
LOC84740	LBR	hsa-mir-184
C20orf26	IGFBP4	hsa-mir-3150b
GDF5	CRISP2	hsa-mir-2355
C2orf40	MIR124-2	hsa-mir-766
SOX11	TNFSF13B	hsa-mir-3199-2
PTCHD1	VIM	hsa-mir-877
SLC6A11	C19orf59	hsa-mir-100
KIF4B	MIR623	hsa-mir-223
KRT16	PCDH8	hsa-mir-585
SLC15A1	MIR190B	hsa-mir-939
TSLP	TNFRSF10A	hsa-mir-129-2
FABP7	TLR1	hsa-mir-1277
SH3GL3	MIR128-1	hsa-mir-615
HORMAD1	LOC285692	hsa-mir-329-2
WDR65	FAM124B	hsa-mir-497
MAPK4	CSF1	hsa-mir-486
MDGA2	HSPB2	hsa-mir-3176
ABCC11	SNORD93	hsa-mir-335
BPI	MT1DP	hsa-mir-3194
TRIML2	SOX9	hsa-mir-488
LEMD1	EGR4	hsa-mir-605
MSLN	VLDLR	hsa-mir-320a
RGS22	NFE2L3	hsa-mir-1301
GJB3	TMEM26	hsa-mir-96

HER2-enriched		
**DNA**	**mRNA**	**miRNA**
LOC100128977	RPS15A	hsa-mir-1307
LY6D	PRAP1	hsa-let-7g
TPSG1	POU4F1	hsa-mir-299
NXNL2	OR11H12	hsa-mir-3909
MAG	PSAT1	hsa-mir-187
OSR1	CR1	hsa-mir-149
LOC100130148	MIR124-2	hsa-mir-3680
SBSN	EGR4	hsa-mir-766
ISL2	NES	hsa-mir-744
CYP4Z2P	PHYHIPL	hsa-mir-127
TUBA3E	ZNF671	hsa-mir-675
SLC6A11	CRYAB	hsa-mir-30c-1
ZIC1	TNFSF13B	hsa-mir-542
CA6	FAM124B	hsa-mir-3926-1
C1QL4	B4GALT6	hsa-mir-550a-1
KRT6C	DARC	hsa-mir-3199-2
CYP2B7P1	NLRP8	hsa-mir-3614
LOC84740	LOC100130331	hsa-mir-100
CXorf61	SOX9	hsa-mir-639
ACE2	SOX2	hsa-mir-136
BPI	TTC36	hsa-mir-153-1
PTPRZ1	CRISP2	hsa-mir-488
C20orf114	TAL2	hsa-mir-335
KRT16	C5orf39	hsa-mir-320a
ROPN1B	DOK5	hsa-mir-3136
WDR65	MIR128-1	hsa-mir-877
C1QL2	PCDH8	hsa-mir-934
RASGEF1C	VIM	hsa-mir-2355
AGR3	IGFBP4	hsa-mir-3150b
MDGA2	OR4N5	hsa-mir-548b
SLC15A1	MIR623	hsa-mir-939
PI3	CSF1	hsa-mir-1301
TMPRSS6	COQ3	hsa-mir-129-2
SOX11	SLC7A5P1	hsa-mir-1229
PTCHD1	ID4	hsa-mir-541
SLC22A16	TLX3	hsa-mir-486
MPP6	EN1	hsa-mir-889
TSLP	NFE2L3	hsa-mir-103-2
C16orf57	LBR	hsa-mir-380
SH3GL3	MIR365-1	hsa-mir-26a-1
RGS22	FLJ35024	hsa-mir-223
C8orf47	C19orf59	hsa-mir-3176
C20orf26	GALT	hsa-mir-944
MSLN	CSTA	hsa-mir-329-2
NCRNA00092	HSPB2	hsa-mir-615
HORMAD1	FLJ41856	hsa-mir-144
C6orf218	TMEM22	hsa-mir-19b-1
MIA	SNORD93	hsa-mir-1277
MGC3771	ZSWIM2	hsa-mir-585
GDF5	TMEM26	hsa-mir-199a-1

Luminal A		
**DNA**	**mRNA**	**miRNA**
LOC100128977	MIR365-1	hsa-mir-3926-1
MAG	RPS15A	hsa-mir-299
TPSG1	NLRP8	hsa-mir-149
LY6D	GALT	hsa-mir-187
ISL2	OR4N5	hsa-let-7g
KRT6C	FLJ35024	hsa-mir-744
CA6	POU4F1	hsa-mir-1307
SBSN	OR11H12	hsa-mir-3680
OSR1	DOK5	hsa-mir-3614
LOC100130148	CR1	hsa-mir-542
NXNL2	B4GALT6	hsa-mir-766
TUBA3E	TAL2	hsa-mir-934
CYP4Z2P	CRYAB	hsa-mir-127
PTPRZ1	PSAT1	hsa-mir-100
ZIC1	C5orf39	hsa-mir-550a-1
SYT9	MIR623	hsa-mir-153-1
C20orf114	IGFBP4	hsa-mir-30c-1
PTCHD1	PCDH8	hsa-mir-3909
CYP2B7P1	FAM124B	hsa-mir-877
SLC22A16	ZNF671	hsa-mir-939
C1QL4	LOC100130331	hsa-mir-3136
KRT16	CRISP2	hsa-mir-380
LOC84740	MIR124-2	hsa-mir-2355
CXorf61	EGR4	hsa-mir-675
RASGEF1C	CSTA	hsa-mir-548b
TMPRSS6	LOC145837	hsa-mir-3199-2
SLC6A11	TTC36	hsa-mir-3150b
WDR65	NFE2L3	hsa-mir-639
SPEF1	GYPC	hsa-mir-486
C20orf26	COQ3	hsa-mir-541
ROPN1B	C19orf59	hsa-mir-1301
MDGA2	PRAP1	hsa-mir-223
ACE2	ID4	hsa-mir-615
KCNK15	HSPB2	hsa-mir-296
AGR3	SOX9	hsa-mir-585
SCN2B	LBR	hsa-mir-1181
SOX11	SOX2	hsa-mir-184
PCSK4	EN1	hsa-mir-497
C1QL2	TLX3	hsa-mir-129-2
SH3GL3	SLC7A5P1	hsa-mir-136
SLC15A1	TNFSF13B	hsa-mir-488
NCRNA00092	NES	hsa-mir-335
TSLP	SNORD93	hsa-mir-320a
FABP7	PHYHIPL	hsa-mir-605
C8orf47	PAPPA2	hsa-mir-376a-2
GDF5	CSF1	hsa-mir-550a-2
MPP6	TNFRSF10A	hsa-mir-3150
HORMAD1	GAS1	hsa-mir-103-2
IGFALS	IRF1	hsa-mir-944
PNMA3	MT1DP	hsa-mir-96

Luminal B		
**DNA**	**mRNA**	**miRNA**
LOC100128977	NLRP8	hsa-mir-3926-1
MAG	GALT	hsa-mir-3680
SBSN	MIR365-1	hsa-mir-299
TPSG1	OR4N5	hsa-mir-187
LY6D	B4GALT6	hsa-let-7g
ISL2	PCDH8	hsa-mir-744
CYP4Z2P	OR11H12	hsa-mir-149
NXNL2	RPS15A	hsa-mir-3614
LOC100130148	EGR4	hsa-mir-1307
OSR1	CRYAB	hsa-mir-766
TUBA3E	POU4F1	hsa-mir-675
KRT6C	GYPC	hsa-mir-542
C1QL4	CRISP2	hsa-mir-3909
CA6	MIR124-2	hsa-mir-934
WDR65	C5orf39	hsa-mir-3136
PTPRZ1	IGFBP4	hsa-mir-30c-1
ZIC1	MIR623	hsa-mir-380
PTCHD1	CR1	hsa-mir-100
CXorf61	DOK5	hsa-mir-153-1
SLC6A11	PSAT1	hsa-mir-127
KRT16	PRAP1	hsa-mir-3199-2
GDF5	LOC100130331	hsa-mir-939
SYT9	ID4	hsa-mir-2355
KCNK15	TAL2	hsa-mir-639
SPEF1	FLJ35024	hsa-mir-877
C20orf114	HSPB2	hsa-mir-1266
ROPN1B	FAM124B	hsa-mir-541
RASGEF1C	LOC145837	hsa-mir-497
TMPRSS6	TTC36	hsa-mir-223
ACE2	SOX9	hsa-mir-550a-1
NCRNA00092	EN1	hsa-mir-376a-2
TSLP	NES	hsa-mir-296
C1QL2	C15orf62	hsa-mir-184
SOX11	ZNF671	hsa-mir-1181
LOC84740	COQ3	hsa-mir-1301
AGR3	PHYHIPL	hsa-mir-136
SH3GL3	SNORD93	hsa-mir-488
SLC15A1	TNFSF13B	hsa-mir-548b
PCSK4	TNFRSF10A	hsa-mir-103-2
C8orf47	TLX3	hsa-mir-3150b
CYP2B7P1	IRF1	hsa-mir-486
SCN2B	C19orf59	hsa-mir-329-2
MPP6	MT1DP	hsa-mir-1229
SLC22A16	LBR	hsa-mir-585
PNMA3	DARC	hsa-mir-335
C2orf40	PAPPA2	hsa-mir-615
RGS20	FLJ41856	hsa-mir-320a
MDGA2	HAS2AS	hsa-mir-3150
HORMAD1	NFE2L3	hsa-mir-129-2
C6orf218	TMEM26	hsa-mir-550a-2

Table 17 Biomarkers with high feature dynamic weights in the KIPAN dataset. We discover the genes that rank in the top 50 for feature dynamic weight in each disease subtype in the KIPAN dataset and highlight those genes with differences as biomarkers.

KICH			KIRC			KIRP		
**meth**	**mRNA**	**miRNA**	**meth**	**mRNA**	**miRNA**	**meth**	**mRNA**	**miRNA**
TRIM58	YWHAB	hsa-mir-20b	AVPR1B	PTPN12	hsa-mir-1306	AVPR1B	SETD3	hsa-mir-1295
ZFP28	COQ9	hsa-mir-3199-2	TRIM58	KLHL3	hsa-mir-342	ZFP28	OAZ2	hsa-mir-3199-2
DGCR10	CDH1	hsa-mir-345	ZFP28	RPL28	hsa-mir-20b	C6orf227	ACLY	hsa-mir-19a
CYP8B1	PSMA7	hsa-mir-598	C6orf227	ABTB2	hsa-mir-605	TRIM58	YWHAB	hsa-mir-605
KLC2	CTDSPL	hsa-mir-10b	STXBP5L	SETD3	hsa-mir-1288	BCAN	NCOA4	hsa-mir-487b
PARP4	PMPCB	hsa-mir-1295	USP4	ANXA2	hsa-mir-487b	MIR219-1	PBLD	hsa-mir-1288
NUPR1	ACLY	hsa-mir-484	DGCR10	CLU	hsa-mir-615	CYP8B1	ANTXR1	hsa-mir-1538
C6orf227	EIF2AK1	hsa-mir-632	NUPR1	PBLD	hsa-mir-1538	C3orf62	GOT1	hsa-mir-337
STXBP5L	ANXA2	hsa-mir-424	CYP8B1	CSDE1	hsa-mir-3199-2	KLC2	PTPN12	hsa-mir-424
USP4	CCL2	hsa-mir-1284	KLC2	EPB49	hsa-mir-10b	USP4	LGALS3	hsa-mir-191
CLDN15	FOXI2	hsa-mir-193a	HSD17B14	EFNB2	hsa-mir-1296	NUPR1	ANXA2	hsa-mir-1296
GUCY2G	ASTN2	hsa-mir-191	NDUFA4L2	ANXA6	hsa-mir-365-1	C6orf58	CXCL9	hsa-mir-615
LRRC37A2	GOT1	hsa-mir-1291	MARCH11	INSR	hsa-mir-3157	IFITM2	GPI	hsa-mir-3130-1
MEIS1	SCOC	hsa-mir-509-2	MIR219-1	LGALS3	hsa-mir-191	NDUFA4L2	EFNB2	hsa-mir-484
MIR23A	STIM1	hsa-mir-337	C6orf58	THY1	hsa-mir-532	C6orf223	TMX2	hsa-mir-345
MIR548F2	SNRPN	hsa-mir-660	IFITM2	LASS2	hsa-mir-19a	STXBP5L	EPB49	hsa-mir-153-2
MIR219-1	ENPEP	hsa-mir-1296	KLF14	ACLY	hsa-mir-146b	GSTM2	FKBP10	hsa-mir-658
GRASP	EPB49	hsa-mir-548s	PARP4	ABCB1	hsa-mir-658	DGCR10	COBLL1	hsa-mir-10b
C3orf62	GAS6	hsa-mir-3157	LOC150786	ALDOA	hsa-mir-484	MIR23A	ANXA6	hsa-mir-1229
OR1J4	CSDE1	hsa-mir-543	C6orf223	LOX	hsa-mir-206	HSD17B14	SMARCE1	hsa-mir-1224
LOC150786	MTSS1	hsa-mir-363	C3orf62	CLDN3	hsa-mir-584	OR1J4	TACC1	hsa-mir-30c-1
NDUFA4L2	CD151	hsa-mir-3934	BCAN	HSP90AB1	hsa-mir-215	PARP4	PMPCB	hsa-mir-584
HSD17B14	CNP	hsa-mir-25	GATA3	YWHAB	hsa-mir-514-2	MEIS1	CCND1	hsa-mir-3944
AVPR1B	IGFBP3	hsa-mir-664	MEIS1	SREBF2	hsa-mir-509-2	MARCH11	HSP90AB1	hsa-mir-1180
IDH2	QARS	hsa-mir-206	IDH2	NCOA4	hsa-mir-1284	LOC150786	LASS2	hsa-mir-215
NRTN	SMARCE1	hsa-mir-1266	GSTM2	OAZ2	hsa-mir-495	CLDN15	COQ9	hsa-mir-20b
IFITM2	TM7SF3	hsa-mir-3150b	RAB6C	C22orf25	hsa-mir-25	BST2	PIK3AP1	hsa-mir-148a
PF4V1	OAZ2	hsa-mir-1306	MIR23A	PMPCB	hsa-mir-337	GATA3	LOX	hsa-mir-1306
CLDN16	METRNL	hsa-mir-1288	NRTN	GOT1	hsa-mir-345	RNF123	ESYT2	hsa-mir-431
RAB6C	KLF6	hsa-mir-3130-1	OR1J4	SMARCE1	hsa-mir-150	PLSCR4	LAPTM5	hsa-mir-382
HTRA4	DMGDH	hsa-mir-3615	PLSCR4	BTF3	hsa-mir-382	KLF14	ETS2	hsa-mir-365-1
KLF14	ATP6V0C	hsa-mir-211	CLDN15	TMCC1	hsa-mir-222	KRTAP3-3	SERPINE2	hsa-mir-139
BCAN	LASS2	hsa-mir-18a	MIR1204	ACTN1	hsa-mir-320d-2	IDH2	CSDE1	hsa-mir-33b
C6orf223	ST5	hsa-mir-30b	DGKE	PRSS23	hsa-mir-450a-1	TACC2	CDK10	hsa-mir-181a-1
RNF123	CXCL9	hsa-mir-584	C1orf230	TMX2	hsa-mir-1180	C1orf230	TFPI	hsa-mir-509-2
MIR1204	TMEM176A	hsa-mir-205	C15orf62	SERINC1	hsa-mir-193a	NRTN	COX6B1	hsa-mir-30d
GSTM2	NDUFV1	hsa-mir-658	CCL5	EIF2AK1	hsa-mir-127	MIR1204	PDK4	hsa-mir-323
FOXL1	RPS13	hsa-mir-301a	BST2	PDK4	hsa-mir-3200	RAB6C	ATP6V0D1	hsa-mir-532
SDS	STRAP	hsa-mir-219-2	GRASP	ESYT2	hsa-mir-1266	IL18BP	ABTB2	hsa-mir-543
C15orf62	PLA2G16	hsa-mir-95	TACC2	LAPTM5	hsa-mir-3130-1	ZNF492	SDPR	hsa-mir-423
MIR886	SREBF2	hsa-mir-1180	NAGS	IDH3A	hsa-mir-212	CCL5	FLII	hsa-mir-548v
NAGS	HSD17B12	hsa-mir-150	SDS	PSMA7	hsa-mir-543	GRASP	PSMA7	hsa-mir-25
GJC2	KHDRBS1	hsa-mir-708	LRRC37A2	CCND1	hsa-mir-181a-1	C15orf62	BTF3	hsa-mir-127
KRTAP3-3	SHISA5	hsa-mir-1224	ZNF492	ACSL1	hsa-mir-632	MIR548F2	TMCC1	hsa-mir-222
GATA3	TMCC1	hsa-mir-2276	MIR886	WDR72	hsa-mir-1295	DGKE	MTSS1	hsa-mir-23a
C5orf4	FBXO7	hsa-mir-148b	CLDN16	PCDH1	hsa-mir-409	PF4V1	SNRPN	hsa-mir-625
COMT	MET	hsa-mir-19a	HTRA4	PROM1	hsa-let-7a-3	LRRC37A2	GLS	hsa-mir-2276
NRG4	BCAR1	hsa-mir-3607	KRTAP3-3	CSF1R	hsa-mir-598	MIR886	ENG	hsa-mir-1284
MARCH11	ST6GAL1	hsa-mir-3189	GUCY2G	UBL3	hsa-mir-3136	RPS6	TOM1	hsa-mir-1266
TACC2	HNRNPK	hsa-mir-139	LGALS9B	TUBA1A	hsa-mir-548o	NAGS	STIM1	hsa-mir-195

Table 18 Biomarkers with high feature dynamic weights in the LGG dataset. We discover the genes that rank in the top 50 for feature dynamic weight in each disease subtype in the LGG dataset and highlight those genes with differences as biomarkers.

Grade 2			Grade 3		
**DNA**	**mRNA**	**miRNA**	**DNA**	**mRNA**	**miRNA**
MIR1469	DBC1	hsa-mir-885	HIST1H4K	DBC1	hsa-mir-3130-1
ZNF714	LRP1B	hsa-mir-3130-1	SNORA37	PER3	hsa-mir-885
LOC644145	PER3	hsa-mir-9-2	MIR1469	LUZP2	hsa-mir-769
NKX2-5	CELF3	hsa-mir-135a-2	NKX2-5	LRP1B	hsa-mir-23c
SNORA37	LUZP2	hsa-mir-769	RFX6	CELF3	hsa-mir-9-2
OR1F1	SESN1	hsa-mir-23c	C14orf139	PDK4	hsa-mir-1225
HIST1H4K	ARHGEF17	hsa-mir-106a	LOC644145	HNRPDL	hsa-mir-668
CSDAP1	HNRPDL	hsa-mir-425	ZNF714	ARHGEF17	hsa-mir-106a
OR6Q1	EIF3L	hsa-let-7a-3	HOXB1	PRNP	hsa-mir-135a-2
HOXB1	PDK4	hsa-mir-185	OR6Q1	C14orf132	hsa-mir-448
RFX6	PRNP	hsa-mir-668	TBX3	EIF3L	hsa-mir-106b
OR9G9	CIRBP	hsa-mir-125b-1	CYP2C18	KIF21B	hsa-mir-876
TBX3	C5orf53	hsa-mir-95	CSDAP1	SESN1	hsa-mir-624
HOXA11AS	LIFR	hsa-mir-876	C1orf54	C5orf53	hsa-mir-95
SNORD88C	C14orf132	hsa-mir-363	OR9G9	CIRBP	hsa-mir-1307
CYP2C18	KIF21B	hsa-mir-1228	OR1F1	LIFR	hsa-mir-150
C14orf139	TMEM100	hsa-mir-651	HOXA11AS	DARS	hsa-let-7a-3
HOXD4	PLCB1	hsa-mir-1307	SNORD88C	TMEM100	hsa-mir-3913-1
C1orf54	SLC22A17	hsa-mir-1225	NPBWR1	PDE4B	hsa-mir-3937
OR2AG1	MATR3	hsa-mir-150	MIR663	ACTB	hsa-mir-641
MIR10B	DARS	hsa-mir-641	MIR10B	APOE	hsa-mir-425
MIR663	SLC39A1	hsa-mir-624	HOXD4	MATR3	hsa-mir-3200
FOXD3	PDE4B	hsa-mir-452	ODAM	SLC22A17	hsa-mir-1251
NPBWR1	AP3B2	hsa-mir-1251	VNN3	PCDH9	hsa-mir-363
ODAM	UQCRB	hsa-mir-106b	SIGLEC11	SKP1	hsa-mir-125b-1
VNN3	APOE	hsa-mir-218-1	PAX9	PLCB1	hsa-mir-452
SIGLEC11	TMEM30A	hsa-mir-375	OR2AG1	U2AF2	hsa-mir-3942
PAX9	U2AF2	hsa-mir-133a-1	FOXD3	DHCR24	hsa-mir-124-1
OR2L3	RTN3	hsa-mir-20a	HLA-DRB6	SLC39A1	hsa-mir-185
FAM75A1	HNRNPH1	hsa-mir-3942	MIR99B	AP3B2	hsa-mir-370
PADI3	SLIT1	hsa-mir-3609	FAM75A1	SLIT1	hsa-mir-20a
HLA-DRB6	ACTB	hsa-mir-136	HOXA6	C4orf3	hsa-mir-375
OR9G1	DAAM2	hsa-mir-579	OR9G1	DAAM2	hsa-mir-133a-1
OR2T2	ACAP3	hsa-mir-3937	LOXL3	GNG5	hsa-mir-216b
FLJ25758	STMN3	hsa-mir-1468	UGT2B28	FAM84B	hsa-mir-561
LOXL3	PDLIM5	hsa-mir-3913-1	OR2L3	BAT2	hsa-mir-627
WDR69	NGFRAP1	hsa-mir-3614	WDR69	CHST1	hsa-mir-579
HOXD13	FAM84B	hsa-mir-378c	PADI3	NGFRAP1	hsa-mir-136
IL23A	BAT2	hsa-mir-9-1	HOXD13	OSGIN2	hsa-mir-9-1
GTF2IRD2B	SET	hsa-mir-27a	FLJ25758	CASC4	hsa-mir-200b
NTS	PCDH9	hsa-mir-214	GTF2IRD2B	H1F0	hsa-mir-30c-1
MIR99B	H1F0	hsa-mir-216b	IL23A	ATPAF1	hsa-mir-491
FGFBP2	PTN	hsa-mir-346	OR2T2	ACAP3	hsa-mir-1228
TPPP3	CHST1	hsa-mir-22	FGFBP2	UQCRB	hsa-mir-7-1
HOXA6	DHCR24	hsa-mir-1249	MIR1247	PTN	hsa-mir-187
HEPHL1	GNG5	hsa-mir-491	NTS	STIP1	hsa-mir-15a
ITPRIPL1	NFE2L2	hsa-mir-561	HEPHL1	LOC643763	hsa-mir-214
MIR1247	SKP1	hsa-mir-30d	DNASE1L3	TBC1D14	hsa-mir-433
BTF3L1	C4orf3	hsa-mir-124-1	ELSPBP1	ENO1	hsa-mir-1468
PSG1	SOX9	hsa-mir-187	BTF3L1	FADS1	hsa-mir-651

Table 19 Biomarkers with high feature dynamic weights in the ROSMAP dataset. We discover the genes that rank in the top 50 for feature dynamic weight in the ROSMAP dataset.

Alzheimer's disease		
DNA methylaton	mRNA	miRNA
ENSG00000188269.3	cg14613972	hsa-miR-450b-5p
ENSG00000203565.2	cg07992625	hsa-miR-1246
ENSG00000142765.12	cg13044136	hsa-miR-767-5p
ENSG00000170759.10	cg27120999	hsa-miR-376c
ENSG00000088836.7	cg10978355	hsa-miR-197
ENSG00000180777.9	cg17692403	hsa-miR-10a
ENSG00000266903.1	cg17826679	hsa-miR-488
ENSG00000253210.1	cg12864235	hsa-miR-491-5p
ENSG00000111087.5	cg01169778	hcmv-miR-UL70-3p
ENSG00000139352.3	cg21663431	hsa-miR-375
ENSG00000254239.1	cg01869233	hsa-miR-640
ENSG00000042062.7	cg20217872	hsv1-miR-H1
ENSG00000267488.1	cg15984661	hsa-miR-432
ENSG00000139629.10	cg25946389	hsa-miR-130b
ENSG00000185499.11	cg12120741	hsa-miR-320a
ENSG00000182851.2	cg06637893	hsa-miR-525-5p
ENSG00000196154.7	cg14837165	hsa-miR-520e
ENSG00000135414.5	cg05382123	hsa-miR-2117
ENSG00000168743.8	cg06933965	hsa-miR-934
ENSG00000154493.12	cg24765079	ebv-miR-BART8
ENSG00000183111.7	cg03258472	hsa-miR-563
ENSG00000198624.8	cg17886959	hsa-miR-518e
ENSG00000230387.1	cg02654291	hsa-miR-1275
ENSG00000177807.5	cg16541031	hsa-miR-135b
ENSG00000171388.9	cg25414165	hsa-miR-34b
ENSG00000183963.13	cg17233506	hsa-miR-770-5p
ENSG00000057704.6	cg11504740	hsa-miR-548a-5p
ENSG00000248714.2	cg07197059	hsa-miR-192
ENSG00000261195.1	cg08727202	hsa-miR-517c
ENSG00000132832.4	cg24101578	hsa-miR-152
ENSG00000165406.9	cg07730301	hsa-miR-487b
ENSG00000188783.5	cg00398048	hsa-miR-651
ENSG00000147488.7	cg17071957	hsa-miR-1180
ENSG00000227392.1	cg24901042	hsa-miR-452
ENSG00000084453.12	cg00777121	hsa-miR-139-5p
ENSG00000166912.12	cg05973262	hsa-miR-296-5p
ENSG00000128271.12	cg14072120	kshv-miR-K12-9
ENSG00000086159.8	cg02293044	hsa-miR-369-3p
ENSG00000140682.14	cg22442730	hsa-miR-10b
ENSG00000166535.15	cg25681177	hsa-miR-27a
ENSG00000254211.1	cg04527918	hsa-miR-93
ENSG00000227544.2	cg09382492	hsa-miR-34a
ENSG00000105227.9	cg08578641	hsa-miR-149
ENSG00000256235.1	cg22398616	hsa-miR-34c-5p
ENSG00000148204.7	cg17253459	hsa-miR-769-5p
ENSG00000235823.1	cg27016307	kshv-miR-K12-2
ENSG00000051128.13	cg19368582	hsa-miR-370
ENSG00000124126.9	cg18636641	hsa-miR-374b
ENSG00000114541.10	cg04983977	hsa-miR-100
ENSG00000134294.9	cg17385448	hsa-miR-891b

Table 20 Comparison of STC1 mRNA expression in COAD and READ. We investigate the expression difference of STC1 between normal and COAD as well as that between normal and READ. Expression alteration is absent in normal and COAD samples but exists in normal and READ samples (p-value = 0.0019).

STC1			
rectal cancer	rectal normal	colon cancer	colon normal
47406	1638.11	6.203369	6.18787
6485.92	1583.47	6.191451	6.386069
13390.3	939.1	6.325832	6.209176
1179.35	941.76	6.103943	6.242934
20404.3	728.74	6.311396	6.126918
441.24	519.79	6.292184	
1839.77	2689.7	6.239597	
5008.26	316.77	6.136832	
6395.17	350.08	6.181462	
4047.49	734.97	6.234069	
14380.7		6.114648	
11046		6.327051	
1401.9		6.466176	
		6.291311	
		6.248177	
		6.199658	
		6.264193	
		6.220789	
		6.154666	
		6.186252	
		6.267337	
		6.27516	
		6.129073	
		6.318915	
		6.304619	
		6.160578	

Table 21 Comparison of STRN4 DNA methylation in COAD and READ. We investigate the expression difference of STRN4 between normal and COAD as well as that between normal and READ. Expression alteration is absent in normal and READ samples but exists in normal and COAD samples (p-value = 0.0363).

	STRN4		
rectal cancer	rectal normal	colon cancer	colon normal
19235.7	22792.7	7.974408	8.100566
13244.2	15180	8.079071	7.992532
8058.47	14104.7	8.274393	7.9126
14214.2	13453	8.136739	7.940537
13900.7	12245.7	8.454869	8.184447
12751.8	11418	8.313291	
17766.7	11124.8	8.311383	
15385.3	11881.5	8.67693	
9855.97	11583.5	8.298457	
23768.3	18446.5	8.563701	
14476.8		8.388082	
10605		8.010227	
26305.1		8.216358	
		8.152823	
		7.998504	
		8.242925	
		8.150253	
		8.25849	
		8.293118	
		8.040017	
		8.337407	
		8.210228	
		8.203544	
		8.039363	
		7.885959	
		7.832084	

3 Supplementary Discussions

Data overview

Pan-cancer dataset. To evaluate the overall performance of our proposed model in predicting pan-cancer, we manually curated a dataset named pan-cancer, which comprises 33 distinct cancer types (as detailed statistics in Supplementary Table 2) sourced from The Cancer Genome Atlas Program (TCGA) (https://www.cancer.gov/ccg/research/genome-sequencing/tcga).
cancer-subtype datasets. We also fetched 12 distinct biomedical datasets for cancer-subtype classification tasks (Supplementary Table 3), which include COADREAD dataset for colon (COAD) and rectal (READ) cancer classification, ESCA dataset for esophageal cancer-subtype classification, GBMLGG dataset for glioblastoma (GBM) and lower-grade glioma (LGG) classification, SARC dataset for sarcoma subtype classification, STAD dataset for stomach adenocarcinoma subtype classification, STES dataset for stomach and esophageal carcinoma subtype classification, THCA dataset for thyroid cancer-subtype classification, UCEC dataset for endometrial cancer for subtype classification, BRCA dataset for breast invasive carcinoma (BRCA) PAM50 subtype classification, KIPAN dataset for kidney cancer-subtype classification, LGG dataset for grade classification in low-grade glioma, ROSMAP dataset for Alzheimer's Disease (AD) patients versus normal control (NC) classification. All abbreviations can be refered to Supplementary Table 7.

Data preprocessing

Given the presence of noise in various datasets and the high dimensionality of DNA methylation and mRNA data, proper preprocessing is crucial for reliable analysis.

On the pan-cancer dataset, we filtered 4,500 significant DNA methylation gene positions and identified 10,180 probes based on the annotation information provided by Illumina (https://www.illumina.com/), then we used the 10,180 probes to overlap with the 450,000-dimensional raw data of methylation to select the significant features. For mRNA, we calculated the standard deviation of each feature and then selected the top 25% features (15165 features) for further analysis.

For the 12 cancer-subtype datasets used in this study (COADREAD, ESCA, GBMLGG, SARC, STAD, STES, THCA, UCEC, BRCA, LGG, KIPAN, and ROSMAP), we adopted a unified preprocessing methodology that entailed dimensionality reduction of DNA methylation and mRNA data via feature selection. Our approach involved filtering out characteristics with no signal (zero mean values) or low variances, with distinct variance thresholds set for different data types (0.1 for mRNA expression and 0.001 for DNA methylation). We then carried out intergroup difference analysis by grouping the data based on classification labels and computing the mean value of each feature for each group. We subsequently evaluated the difference between the mean values

of each feature across the groups and retained the top 2000 features displaying the highest mean value differences across groups. These features served as the preprocessed datasets for DNA methylation and mRNA respectively. However, since miRNA data has a limited number of available features, we didn't filter any features except if lacking signal (zero mean values), but rather utilized the original data exclusively for the experiment. Finally, we scaled each type of omic data independently to [0, 1] during training using the MinMaxScaler.

Training details

During the course of our training, it was deemed imperative to implement the 5-fold cross-validation training strategy, which allowed us to derive the average and standard deviation of the 5 test results on the validation set for each dataset. The aforementioned approach, while preventing overfitting to a great extent, was also instrumental in providing a more accurate and reliable evaluation of the model's performance. Table **??** and Supplementary Table 3 list the results of all classification tasks.

In an effort to delve deeper into the individualized contribution of each module to HTML model, a series of ablation studies were conducted concerning the COADREAD dataset. As expected, results revealed that all modules played an indispensable role in HTML, with the removal of the methylation-guided attention module causing a drop in accuracy by approximately 10%, and the removal of the dynamic learning modules causing a drop in accuracy by approximately 5%. Supplementary Table 4 illustrates the detailed results of these findings.

In order to identify a set of hyper-parameters that would yield the optimal performance for each dataset, we employed the grid search strategy. This enabled us to identify and recommend the most suitable hyper-parameters, including the hidden dimension, training epoch, dropout rate, weight decay, L1 lambda and learning rate. Supplementary Table 5 provides a comprehensive listing of these recommended hyper-parameters.

Biomarker identification with HTML

The feature dynamic module provides the weight of each feature, thereby enabling the identification of biomarkers for specific cancer and cancer-subtypes. Features with higher feature dynamic weights are particularly significant in identifying biomarkers. In our study, we first calculated the average feature dynamic weight of a specific DNA methylation spot, mRNA, and miRNA. We then sorted all the weights of the features and identified the top 50 high-weighted features of each modality. Finally, we compared the differences in biomarkers corresponding to different types of cancer and selected the differential biomarkers as the reported results. This procedure resulted in a more targeted selection of potential biomarkers for further exploration in the analysis of specific cancer types and subtypes.

Biomarkers divergent in individuals and cancer types

Biomarkers have emerged as valuable tools for identifying both individual characteristics and cancer types. Although some biomarkers may serve both purposes, there are often discrepancies in the set of biomarkers used for individual versus cancer type identification [7]. Our experiments reveal differences in the biomarkers identified for individual diagnosis and cancer sub-type identification.

For instance, in our analysis of the 66th sample (Fig. 2c), we identified HNF1A DNA methylation, MARK4 mRNA expression, and hsa-mir-1307 miRNA expression as the top biomarkers for individual diagnosis. However, in the identification of biomarkers for the esophageal squamous cell carcinoma (ESCC) sub-type, the HNF1A DNA methylation ranked 56th among the DNA methylation biomarkers, the MARK4 mRNA expression ranked 38th among the mRNA expression biomarkers, and the hsa-mir-1307 miRNA expression ranked 13th among the miRNA biomarkers. These results highlight the significant difference between individual biomarker analysis and overall cancer sub-type biomarkers.

Undoubtedly, the identification of individual biomarkers can bring higher precision medicine values in personalized treatment. Individual biomarkers are utilized to identify specific biological characteristics of an individual, such as genetic variations, that may impact their health or response to treatment. In contrast, cancer biomarkers are used to identify specific characteristics of a particular type of cancer. These biomarkers can facilitate cancer diagnosis, progression monitoring, and the development of targeted therapies. Our HTML model proposes a highly trustworthy and sample adaptive model that can benefit the entire chain of cancer research and diagnosis.

Comparison with statistical methods

Both data-driven statistical methods and HTML model can be used for discovering biomarkers. However, there are differences between the two methods. Data-driven statistical methods typically rely on previous research results and use extensive statistical analyses to identify biomarkers. In contrast, HTML model is an adaptive model that can adjust to the sample, allowing for more accurate biomarker discovery.

HTML model can simultaneously identify individual and cancer sub-type biomarkers with higher reliability. In comparison, data-driven statistical methods typically only identify a specific type of biomarker, which may overlook important information related to other biomarkers and other omics. Therefore, HTML model has broader application prospects in cancer research and diagnosis, and can improve the accuracy and effectiveness of cancer diagnosis and treatment.

References

[1] Leo Breiman. Random forests. *Machine learning*, 45:5–32, 2001.

[2] Jonathan Buckley and Ian James. Linear regression with censored data. *Biometrika*, 66(3):429–436, 1979.

[3] Tianqi Chen and Carlos Guestrin. Xgboost: A scalable tree boosting system. In *Proceedings of the 22nd acm sigkdd international conference on knowledge discovery and data mining*, pages 785–794, 2016.

[4] Yizeng Han, Gao Huang, Shiji Song, Le Yang, Honghui Wang, and Yulin Wang. Dynamic neural networks: A survey. *IEEE Transactions on Pattern Analysis and Machine Intelligence*, 44(11):7436–7456, 2021.

[5] Zongbo Han, Fan Yang, Junzhou Huang, Changqing Zhang, and Jianhua Yao. Multimodal dynamics: Dynamical fusion for trustworthy multimodal classification. In *Proceedings of the IEEE Conference on Computer Vision and Pattern Recognition*, pages 20707–20717, 2022.

[6] Kevin P Murphy et al. Naive bayes classifiers. *University of British Columbia*, 18(60):1–8, 2006.

[7] Virinder Kaur Sarhadi and Gemma Armengol. Molecular biomarkers in cancer. *Biomolecules*, 12(8):1021, 2022.

[8] Roman Schulte-Sasse, Stefan Budach, Denes Hnisz, and Annalisa Marsico. Integration of multiomics data with graph convolutional networks to identify new cancer genes and their associated molecular mechanisms. *Nature Machine Intelligence*, 3(6):513–526, 2021.

[9] Johan AK Suykens and Joos Vandewalle. Least squares support vector machine classifiers. *Neural processing letters*, 9:293–300, 1999.

[10] Tongxin Wang, Wei Shao, Zhi Huang, Haixu Tang, Jie Zhang, Zhengming Ding, and Kun Huang. Mogonet integrates multi-omics data using graph convolutional networks allowing patient classification and biomarker identification. *Nature communications*, 12(1):3445, 2021.

[11] Jing Zhao, Bowen Zhao, Xiaotong Song, Chujun Lyu, Weizhi Chen, Yi Xiong, and Dong-Qing Wei. Subtype-dcc: decoupled contrastive clustering method for cancer subtype identification based on multi-omics data. *Briefings in Bioinformatics*, 24(2):bbad025, 2023.
